# Choosing Optimal Antibiotics for the Treatment of Patients Infected With *Enterobacteriaceae*: A Network Meta-analysis and Cost-Effectiveness Analysis

**DOI:** 10.3389/fphar.2021.656790

**Published:** 2021-06-17

**Authors:** Ruiying Han, Mengmeng Teng, Ying Zhang, Tao Zhang, Taotao Wang, Jiaojiao Chen, Sihan Li, Bo Yang, Yaling Shi, Yalin Dong, Yan Wang

**Affiliations:** ^1^ Department of Pharmacy, The Second Affiliated Hospital of Xi’an Jiaotong University, Xi’an, China; ^2^ Department of Pharmacy, The First Affiliated Hospital of Xi’an Jiaotong University, Xi’an, China; ^3^ School of Pharmacy, Xi’an Jiaotong University, Xi’an, China; ^4^ Department of Pharmacy, Shaanxi Provincial People’s Hospital, Xi’an, China

**Keywords:** *Enterobacteriaceae*, network meta-analysis, complicated urinary tract infection, novel β-lactam/β-lactamase inhibitors, cost-effectiveness analysis

## Abstract

Overuse of carbapenems has led to the increasing carbapenem-resistant *Enterobacteriaceae*. It is still unknown whether other antibiotics [especially novel *β*-lactam/β-lactamase inhibitor combinations (BL/BLIs)] are better than carbapenems in the treatment of *Enterobacteriaceae*. A systematic literature search was performed to identify randomized controlled trials (RCTs) assessing the efficacy and safety of any antibiotics on *Enterobacteriaceae* infections. We carried out a traditional paired meta-analysis to compare ceftazidime/avibactam to comparators. Network meta-analysis (NMA) was conducted to integrate direct and indirect evidence of all interventions. Moreover, cost-effectiveness analysis using a combined decision analytical Markov model was completed for the treatment of patients with complex urinary tract infection (cUTI). A total of 25 relevant RCTs were identified, comprising 15 different interventions. Ceftazidime/avibactam exhibited comparable efficacy and safety with comparators (carbapenems) in the paired meta-analysis. In the NMA, the surface under the cumulative ranking curve probabilities showed that in terms of efficacy, the interventions with the highest-ranking were meropenem/vaborbactam, meropenem, imipenem/cilastatin, ceftriaxone, ceftazidime/avibactam, and ceftolozane/tazobactam [but no significant difference between any two antibiotics (*p* > 0.05)]. Regarding safety, ceftazidime/avibactam had a higher incidence of adverse events than that of piperacillin/tazobactam (relative risk = 0.74, 95% confidence interval = 0.59–0.94). Based on drug and hospitalization costs in China, the incremental cost-effectiveness ratio per quality-adjusted life-year gained in the patients with cUTI for meropenem, ceftazidime/avibactam, and ceftolozane/tazobactam compared to imipenem/cilastatin were US$579, US$24569, and US$29040, respectively. The role of these BL/BLIs to serve as alternatives to carbapenems requires large-scale and high-quality studies to validate.

## Introduction

*Enterobacteriaceae* infections are major types of hospital-acquired infections. Carbapenems are the first-line antibiotics for the treatment of multidrug-resistant *Enterobacteriaceae* infections, but long-term overuse has gradually increased the resistance of *Enterobacteriaceae* to them (Temkin et al., 2014). A study in 2001 first reported that *Klebsiella pneumoniae* showed moderate to high resistance to imipenem and meropenem (Yigit et al., 2001). Since then, carbapenem-resistant *Enterobacteriaceae* (CRE) has spread globally (Tängdén and Giske, 2015).

Appropriate initial empirical therapy is particularly important in the challenging drug-resistance situation, where improper use of antibiotics increases mortality and economic burden for patients (Girometti et al., 2014; Zilberberg et al., 2017; Tacconelli et al., 2019). Current antibiotics available for *Enterobacteriaceae* infections include *β*-lactam/β-lactamase inhibitors (BL/BLIs) and other existing antibiotics (e.g., aminoglycosides, carbapenems, tigecycline, etc.). The clinical efficacy of novel BL/BLIs (e.g., ceftazidime/avibactam, ceftolozane/tazobactam, and meropenem/vaborbactam) in the treatment of *Enterobacteriaceae* infections have been well studied (Solomkin et al., 2015; Carmeli et al., 2016; Wunderink et al., 2018), but there are few other-antibiotics-related studies directly compared to them. Although there has been some meta-analysis on the choice of antibiotics used for *Enterobacteriaceae* infections (Sfeir et al., 2018; Son et al., 2018; Che et al., 2019), the relative efficacy of existing treatments is still uncertain (especially between novel BL/BLIs with standard-of-care treatment or among different carbapenems) due to the lack of direct comparisons among many antibiotics. In summary, the lack of direct evidence and indirect evidence makes it difficult to obtain the hierarchy for antibiotics to treat *Enterobacteriaceae* infections, and it is unclear whether there are more effective initial treatment strategies.

In the real world where antibiotics resistance is rising, traditional clinical analyses do not reflect the true value of antibiotics because they often exclude suspected drug-resistant patients and adopt a non-inferiority design. However, it is known that the resistance rate of *Enterobacteriaceae* to many antibiotics is relatively high in China (Zhang et al., 2018). Pharmacoeconomics evaluation is particularly important for antibiotics, especially for novel agents, to help identify their true market value in the presence of therapeutic resistance (Verhoef and Morris, 2015; Naylor et al., 2018).

Hence, this study aimed to integrate the available direct and indirect evidence *via* network meta-analysis (NMA) to comprehensively assess the clinical efficacy and safety of any antibiotics for the treatment of *Enterobacteriaceae* infections. We also developed a pharmacoeconomic analysis model to evaluate the cost-effectiveness of antibiotics from the perspective of healthcare setting.

## Methods

### Selection Criteria

The following inclusion criteria were applied: 1) randomized controlled trials (RCTs); 2) adult patients (≥18 years); 3) evaluating the therapeutic effect of any antibiotics on *Enterobacteriaceae* infections; 4) reporting at least one outcome of the clinical success, the microbiological success, the incidence of adverse events, or mortality data; 5) infection by *Enterobacteriaceae* ≥80% of the whole population. Studies were excluded if they met the following criteria: 1) meta-analysis, letters, reviews, case reports, or editorial comments; 2) combined therapy study; 3) full text not available.

### Search Strategy

RCTs were searched from PubMed, embase, Cochrane Library databases, and ClinicalTrials.gov, starting from their inception to May 2020. The detailed search strategy was in Supplementary Appendix S1. In addition, manual searches were performed in the reference lists of all included articles and related review studies to obtain possibly eligible trials.

### Study Selection and Data Extraction

Two researchers independently screened the literatures and excluded studies that did not meet the inclusion criteria by reading the titles and abstracts. After the initial screening, the full-text articles that fulfilled requirements were screened to further determine whether to include. The inconsistencies between researchers were resolved through negotiation. If no agreement was reached, the third investigator decided whether to include the controversial study.

The following data were extracted: authors, publication year, study design, countries, patient characteristics (age, sex, the proportion of *Enterobacteriaceae*, infections type), treatment regimen, outcomes, and financial support. The primary outcomes were the clinical success (i.e., the signs and symptoms of infections were completely disappeared or significantly improved without further antibiotics treatment) and the microbiological success (defined by each RCT); the safety outcomes (the incidence of adverse events and mortality) were assessed as secondary outcomes.

The risk of bias of the included literatures in this systematic review was appraised by the Cochrane Risk of Bias Tool.

### Statistical Analysis

A paired meta-analysis based on the random-effects model was used to compare ceftazidime/avibactam (the only novel BL/BLI marked in China) with comparators. The estimates of primary and secondary outcomes were determined using relative risk (RR) and their corresponding 95% confidence interval (CI). A *p* value of <0.05 was considered statistically significant. In this study, *I* ^2^ statistics was used to evaluate heterogeneity quantitatively. If *I* ^2^ > 50%, it indicated that there was significant heterogeneity between the studies. Besides, subgroups of different infection types [i.e., complicated urinary tract infection (cUTI) and complex intra-abdominal infection (cIAI)] were planned to examine the impact on outcomes.

We applied Mvmeta command in Stata (version 15.1) software to conduct random-effects NMA, assessing the efficacy and safety of different interventions through direct and indirect comparisons (Caldwell et al., 2005; Cipriani et al., 2013). RR and their corresponding 95% CI were used to evaluate the effect of various antibiotics.

The surface under the cumulative ranking curve (SUCRA) probabilities were reported for primary outcomes to estimate the treatment rank for all interventions (Salanti et al., 2011; Chaimani et al., 2013). Moreover, the ranks of the clinical success and the microbiological success were added together to comprehensively appraise the efficacy. Interventions with the same ranking would be re-ranked based on the sum of their SUCRA probabilities. A common estimate [tau (τ) value] was used to assess the heterogeneity between studies and the degree of heterogeneity was evaluated by comparing the value of *τ*(Turner et al., 2012). We conducted publication bias by drawing funnel plots for the primary outcomes. In addition, we used the inconsistency model to analyze the consistency of the studies. If *p* < 0.05, it indicated that the inconsistency model was statistically significant and the consistency model cannot be used for analysis (Song et al., 2012; Sturtz and Bender, 2012). Furthermore, a subgroup analysis was conducted to evaluate the impact of different infection types (i.e., cUTI and cIAI) on the efficacy of antibiotics.

We carried out sensitivity analyses of the primary outcomes to determine the stability of the outcomes by excluding RCTs with more than one item indicating a high-risk bias, and excluding RCTs with a sample size of less than 100 and 30.

### Cost-Effectiveness Analysis

Given that the best available evidence for patients with cUTI (more close-loop formed), we only chose this infection type for economic evaluation.

A combined decision analytical Markov model was constructed to estimate the cost-effectiveness of initiating empirical antibiotics treatment for cUTI patients caused by *Enterobacteriaceae*, using the software of TreeAge Pro 2011 (TreeAge Software, Inc., MA, United States) (Supplementary Appendix Figure S1). Patients entered the model at the time of cUTI diagnosis and showed the presence of *Enterobacteriaceae*. The clinical success was derived from the meta-analysis of this study. The data of other variables and cost were obtained from published literatures or government data (Supplementary Appendix Table S1 and Supplementary Appendix Figures S2–S5). Total costs and quality-adjusted life years (QALY) gained were estimated. The incremental cost-effectiveness ratios (ICERs) per additional QALY gained was calculated to compare the performance of treatment strategies. The potential influence of variations of key parameters on ICERs was investigated by deterministic and probabilistic sensitivity analyses (Supplementary Appendix S2).

## Results

### Characteristics of Included Trials

**FIGURE 1:**
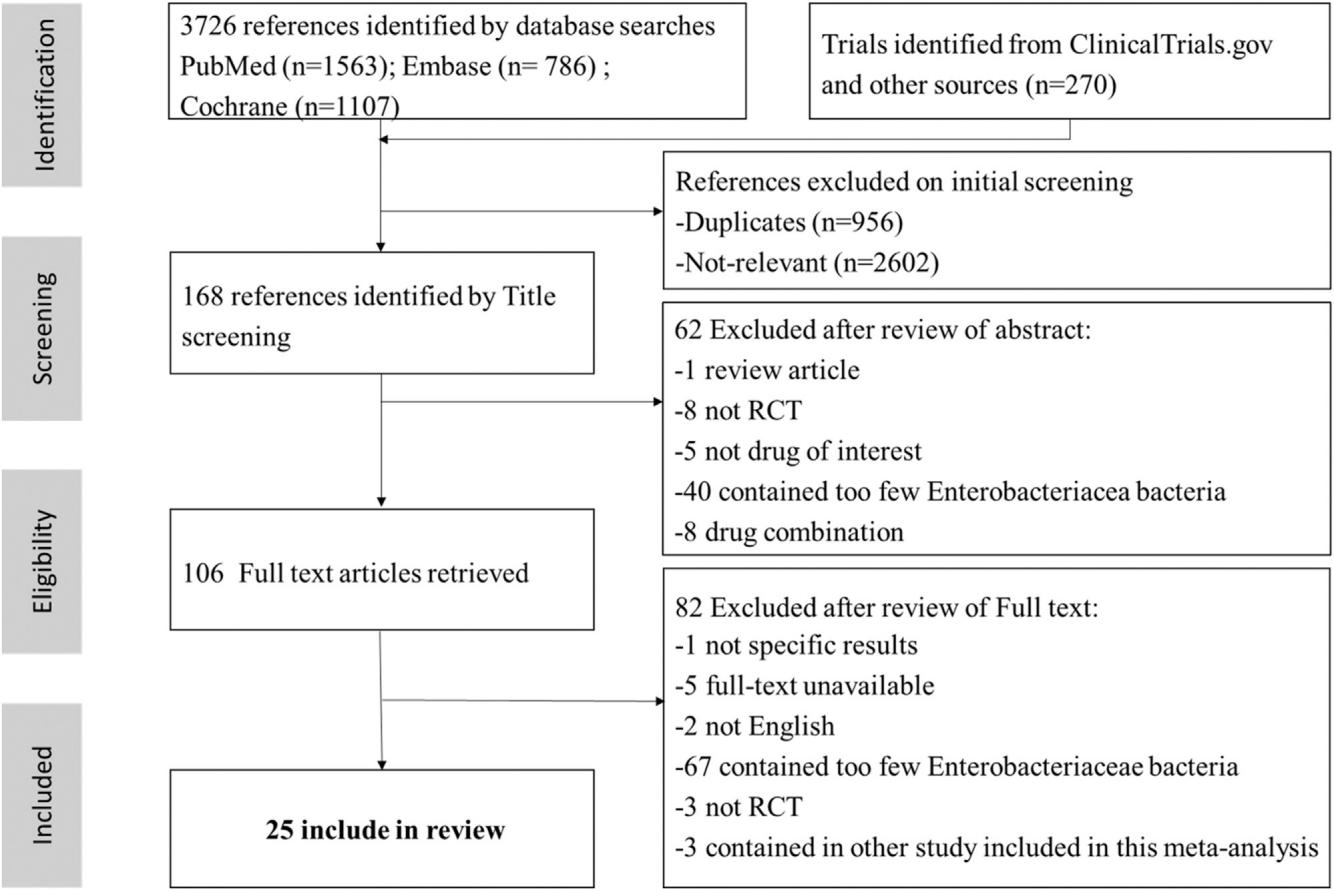
Study selection flow diagram.

A total of 3,726 articles were initially retrieved. 3,620 articles were excluded through preliminary screening, and the remaining 106 articles were chosen for full text reading. Finally, twenty-five RCTs of 15 antibiotics were selected out (Figure 1) (Sifuentes-Osornio et al., 1989; Preheima et al., 1995; Chang et al., 1998; Richard et al., 1998; Ponce-de-León et al., 1999; Jimenez-Cruz et al., 2002; Tomera et al., 2002; Erasmo et al., 2004; Wells et al., 2004; Klausner et al., 2007; Fomin et al., 2008; Naber et al., 2009; Ceran et al., 2010; Park et al., 2012; Vazquez et al., 2012; Solomkin et al., 2015; Wagenlehner et al., 2015; Carmeli et al., 2016; Mazuski et al., 2016; Wagenlehner et al., 2016; Qin et al., 2017; Seo et al., 2017; Harris et al., 2018; Kaye et al., 2018; Kaye et al., 2019).

**FIGURE 2:**
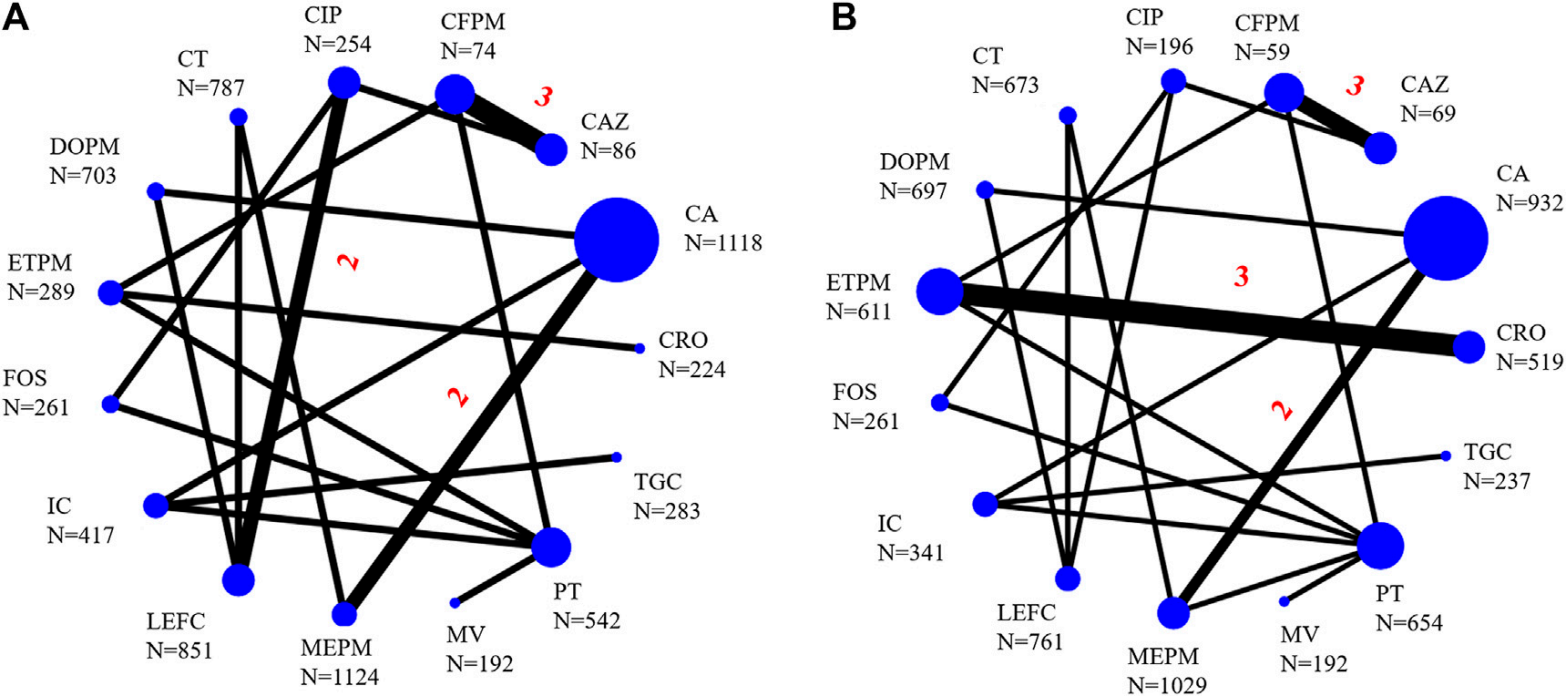
Network of eligible comparisons for primary outcomes [**(A)**, clinical success; **(B)**, microbiological success]. Straight-line represented direct comparisons of antibacterial drugs, the thickness of which corresponded to the number of included studies, the unmarked number means there was only one head-to-head comparison. N was the sample size corresponding to the antibiotics. CA, ceftazidime/avibactam; MV, meropenem/vaborbactam; MEPM, meropenem; DOPM, doripenem; IC, imipenem/cilastatin; CAZ, ceftazidime; CFPM, cefepime; CIP, ciprofloxacin; PT, piperacillin/tazobactam; ETPM, ertapenem; LEFC, levofloxacin; FOS, fosfomycin; CT, ceftolozane/tazobactam; TGC, tigecycline; CRO, ceftriaxone.

The infection types of 25 RCTs were cIAI (*n* = 4, 16%) (Erasmo et al., 2004; Solomkin et al., 2015; Mazuski et al., 2016; Qin et al., 2017), cUTI (*n* = 17, 68%) (Preheima et al., 1995; Richard et al., 1998; Jimenez-Cruz et al., 2002; Tomera et al., 2002; Wells et al., 2004; Klausner et al., 2007; Fomin et al., 2008; Naber et al., 2009; Ceran et al., 2010; Park et al., 2012; Vazquez et al., 2012; Wagenlehner et al., 2015; Wagenlehner et al., 2016; Seo et al., 2017; Harris et al., 2018; Kaye et al., 2018; Kaye et al., 2019), mixed infection types (*n* = 3, 12,5%) (Sifuentes-Osornio et al., 1989; Chang et al., 1998; Ponce-de-León et al., 1999) [the remaining RCT included both cIAI and cUTI patients (Carmeli et al., 2016)]. A total of 10,390 participants were involved in this study, and the main characteristics of each study were summarized in Supplementary Appendix Table S2 in Supplementary Appendix S3. Twenty-four RCTs were included in the NMA [one study was not included because it did not classify different carbapenems (Carmeli et al., 2016)], and the networks of eligible comparisons for primary outcomes were presented in Figure 2. Five of twenty-five RCTs related to ceftazidime/avibactam were included in the paired meta-analysis (Vazquez et al., 2012; Carmeli et al., 2016; Mazuski et al., 2016; Wagenlehner et al., 2016; Qin et al., 2017), and all the comparators were carbapenems (meropenem, doripenem, or imipenem/cilastatin).

About half of the trials provided detailed procedures for sequence generation (15 RCTs, 60%) and allocation sequence concealment (14 RCTs, 56%). Five trials were open-label studies with high risks for performance bias and detection bias (Chang et al., 1998; Ponce-de-León et al., 1999; Erasmo et al., 2004; Seo et al., 2017; Harris et al., 2018). One trial was a single-blind experiment with high risks for performance bias (Ceran et al., 2010). Two studies had missing data with high risks for attrition bias (Klausner et al., 2007; Ceran et al., 2010) (Supplementary Appendix Figures S6, S7 in Supplementary Appendix S4).

### Meta-Analysis

#### Efficacy

**FIGURE 3:**
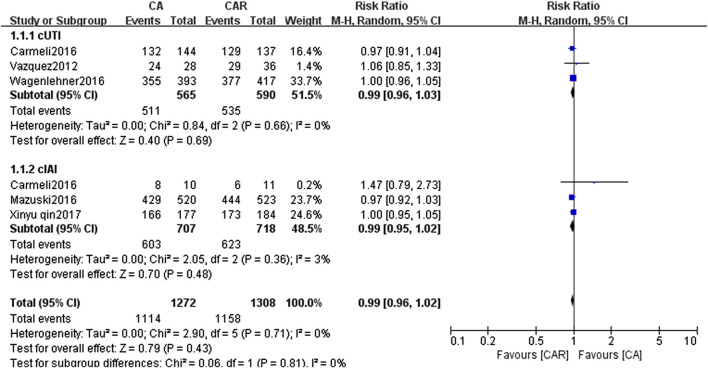
Forest plots showing relative risk with 95% confidence interval of clinical success in a random-effects model. cUTI, complex urinary tract infection; cIAI, complicated intra-abdominal infection; CA, ceftazidime/avibactam; CAR, carbapenems.

**FIGURE 4:**
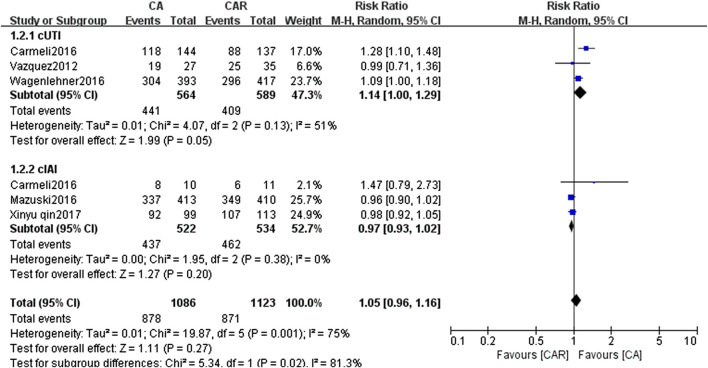
Forest plots showing relative risk with 95% confidence interval of microbiological success in a random-effects model. cUTI, complex urinary tract infection; cIAI, complicated intra-abdominal infection; CA, ceftazidime/avibactam; CAR, carbapenems.

The results of paired meta-analyses showed that there was no statistically significant difference between ceftazidime/avibactam and the comparators (carbapenems) in the clinical success and microbiological success (RR = 0.99, 95% CI = 0.96–1.02; RR = 1.05, 95% CI = 0.96–1.16, respectively) (Figures 3, 4). Similarly, no significant difference was detected in cUTI and cIAI subgroups.

**FIGURE 5:**
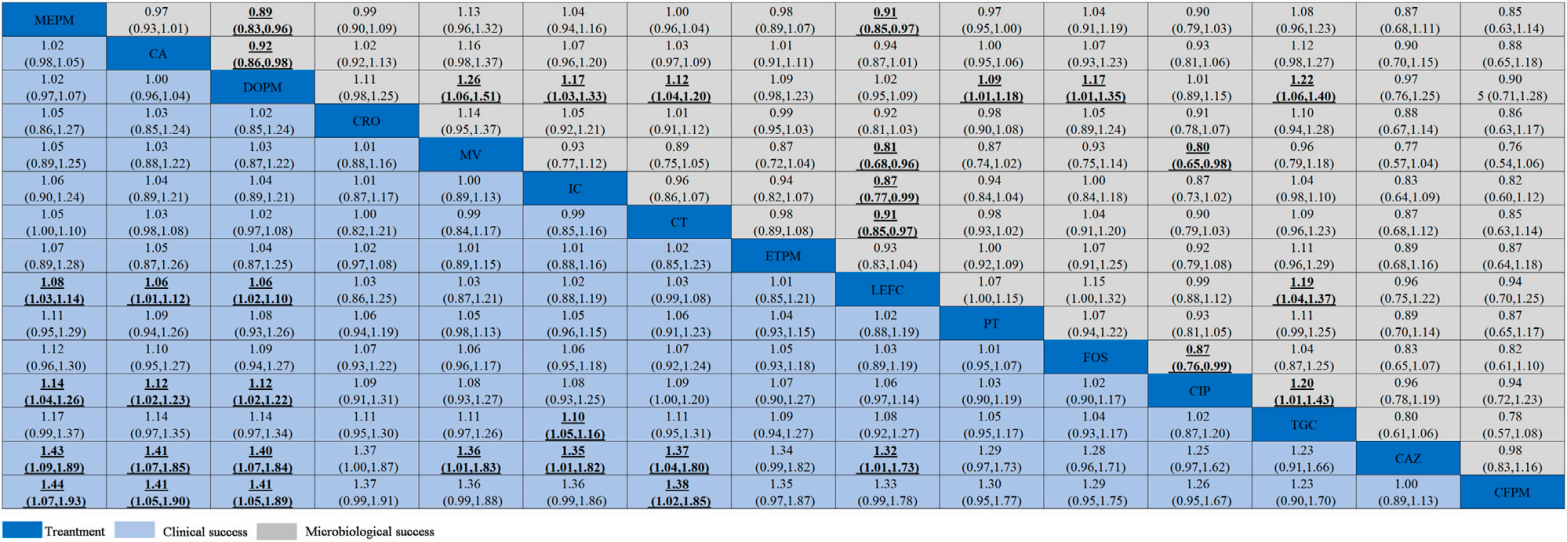
Clinical success and microbiological success of interventions in the treatment of *Enterobacteriaceae* infections. Antibiotics were sorted in the order of decreasing clinical cure rate. The clinical success results were compared from left to right, and the microbiological success results should be read from right to left (the results were expressed by relative risk with 95% confidence interval). Significant results were shown in bold and underlined. CA, ceftazidime/avibactam; MV, meropenem/vaborbactam; MEPM, meropenem; DOPM, doripenem; IC, imipenem/cilastatin; CAZ, ceftazidime; CFPM, cefepime; CIP, ciprofloxacin; PT, piperacillin/tazobactam; ETPM, ertapenem; LEFC, levofloxacin; FOS, fosfomycin; CT, ceftolozane/tazobactam; TGC, tigecycline; CRO, ceftriaxone.

In the NMA, the clinical success of meropenem was significantly more effective than levofloxacin (RR = 1.08, 95% CI = 1.03–1.14), ciprofloxacin (RR = 1.14, 95% CI = 1.04–1.26), ceftazidime (RR = 1.43, 95% CI = 1.09–1.89), and cefepime (RR = 1.44, 95% CI = 1.07–1.93); ceftazidime/avibactam was statistically superior than levofloxacin (RR = 1.06, 95% CI = 1.01–1.12), ciprofloxacin (RR = 1.12, 95% CI = 1.02–1.23), ceftazidime (RR = 1.41, 95% CI = 1.07–1.85), and cefepime (RR = 1.41, 95% CI = 1.05–1.9) (Figure 5). As for microbiological success, doripenem was significantly lower than that of meropenem (RR = 0.89, 95% CI = 0.83–0.96) and ceftazidime/avibactam (RR = 0.92, 95% CI = 0.86–0.98). Levofloxacin was also observed to be significantly less effective than meropenem (RR = 0.91, 95% CI = 0.85–0.97) (Figure 5).

We used SUCRA probabilities to display the rank order for all interventions (Table 1). In the clinical success, the interventions with the highest-ranking were (*via* SUCRA probabilities): meropenem (87.9), ceftazidime/avibactam (77.7), doripenem (75.7), ceftriaxone (67.7), meropenem/vaborbactam (66.2), imipenem/cilastatin (63.6), and ceftolozane/tazobactam (63.5). Meropenem/vaborbactam (90.6) ranked first on the microbiological success, followed by tigecycline (87.7), imipenem/cilastatin (74.6), fosfomycin (74.4), meropenem (64.4), ceftolozane/tazobactam (61.8), and ceftriaxone (57.7). After integrative assessments (i.e. adding the rankings of the clinical success and the microbiological success together), antibiotics ranked first to seventh were meropenem/vaborbactam, meropenem, imipenem/cilastatin, ceftriaxone, ceftazidime/avibactam, ceftolozane/tazobactam, and tigecycline. Nevertheless, there is no significant difference between the clinical success and microbiological success of these interventions. The funnel plot showed no asymmetry (Supplementary Appendix Figure S8 in Supplementary Appendix S5).

**TABLE 1:** Results of SUCRA in primary and secondary outcomes for *Enterobacteriaceae* infections.

Treatment	Clinical success		Microbiological success		Overall rank^a^	Adverse events		Mortality	
	SUCRA	Rank	SUCRA	Rank		SUCRA	Rank	SUCRA	Rank
Meropenem/vaborbactam	66.2	5	90.6	1	1	66	3	40.9	11
Meropenem	87.9	1	64.4	5	2	45.8	8	77.4	1
Imipenem/cilastatin	63.6	6	74.6	3	3	18.5	11	75.6	2
Ceftriaxone	67.7	4	57.7	7	4	—	—	39.9	12
Ceftazidime/avibactam	77.7	2	44.7	10	5	28.4	10	60.0	5
Ceftolozane/tazobactam	63.5	7	61.8	6	6	43.9	9	61.7	4
Tigecycline	23.8	13	87.7	2	7	16.2	12	—	—
Fosfomycin	36.7	11	74.4	4	8	43.9	9	43.9	8
Ertapenem	57.6	8	49.2	8	9	—	—	45.7	7
Doripenem	75.7	3	15	15	10	50.3	5	59.7	6
Piperacillin/tazobactam	41.1	10	45.5	9	11	82	1	37.5	13
Levofloxacin	46.6	9	19.9	14	12	60.8	4	63.8	3
Ciprofloxacin	28.9	12	22.2	11	13	67.5	2	42.8	9
Ceftazidime	6.5	14	21.9	12	14	47.9	6	41.0	10
Cefepime	6.5	15	20.4	13	15	47.6	7	10.1	14

SUCRA, the surface under the cumulative ranking curve. The orders of clinical success and microbiological success were from best to worst, while the adverse events and mortality were from lowest to highest.

a adding the order of clinical success and microbiological success together (Interventions with the same ranking would be re-ranked based on the sum of their SUCRA probabilities).

In subgroup analysis, with respect to cUTI, thirteen antibiotics except meropenem were ranked by SUCRA probabilities (the only included RCT on meropenem did not report the clinical success), and we found that the clinical success of doripenem was significantly higher than that of levofloxacin (RR = 1.05, 95% CI = 1.01–1.11) and ciprofloxacin (RR = 1.11, 95% CI = 1.01–1.11). As to the microbiological success, ceftolozane/tazobactam was more effective than that of doripenem (RR = 1.13, 95% CI = 1.01–1.26), and other detailed results were shown in Supplementary Appendix Figure S9 of Supplementary Appendix S6. Based on the integrative assessments of efficacy, the antibiotics with the highest SUCRA probability were ceftolozane/tazobactam, followed by ceftazidime/avibactam, doripenem, meropenem/vaborbactam, and imipenem/cilastatin (Supplementary Appendix Table S3 in Supplementary Appendix S6).

#### Safety

No statistically significant difference between ceftazidime/avibactam and comparators for the incidence of adverse events and mortality in paired meta-analysis was noticed (RR = 1.01, 95% CI = 0.90–1.12; RR = 1.40, 95% CI = 0.69–2.85, respectively) (Supplementary Appendix Figures S10, S11 in Supplementary Appendix S7). As to the subgroup analysis of different infection types, the results showed that the incidence of adverse events and mortality were similar between the two subgroups in cUTI and cIAI.

In the NMA, ceftriaxone and ertapenem were excluded in the analysis of the incidence of adverse events because they cannot be directly compared with other antibiotics. The incidence of adverse events of piperacillin/tazobactam was lower than that of ceftazidime/avibactam (RR = 0.74, 95% CI = 0.59–0.94) and imipenem/cilastatin (RR = 0.68, 95% CI = 0.53–0.87) (Supplementary Appendix Figure S12 in Supplementary Appendix S7). Regarding mortality, tigecycline was not analyzed because it led to 0 deaths in each study. Notably, ceftazidime/avibactam demonstrated lower mortality than that of cefepime (RR = 0.12, 95% CI = 0.02–0.86); the mortality of meropenem was less frequent than that of piperacillin/tazobactam and cefepime (RR = 0.31, 95% CI = 0.14–0.68; RR = 0.07, 95% CI = 0.01–0.46, respectively). The SUCRA probabilities indicated that piperacillin/tazobactam has the lowest incidence of adverse events, and the mortality of cefepime was the highest (Table 1).

#### Assessment of Heterogeneity and Inconsistency

The result of the microbiological success showed moderate heterogeneity (*I* ^2^ = 75%), while the remaining outcomes (the clinical success, the incidence of adverse events and mortality, respectively) had no significant heterogeneity in the paired meta-analysis (*I* ^2^ = 0%, *I* ^2^ = 32%, *I* ^2^ = 0%, respectively). In the NMA, the heterogeneity of clinical success, microbiological success, the incidence of adverse events and mortality were τ = 3.841e^−9^, τ = 5.662e^−11^, τ = 1.908e^−9^, τ = 3.521e^−9^, respectively, indicating low heterogeneity on all outcomes. Additionally, the clinical success, the microbiological success, the incidence of adverse events and mortality displayed no inconsistency in the inconsistency model analysis (*p* = 0.56; *p* = 0.22; *p* = 0.40; *p* = 0.67, respectively).

#### Sensitivity Analysis for Network Meta-Analysis

In the two pre-designed sensitivity analyses, since too many antibiotics in low-risk RCTs cannot form a closed loop with other antibiotics, the studies with a sample size of more than 30 and 100 were analyzed. The results showed no significant change compared with the original result, suggesting that the results were robust (Supplementary Appendixes S8, S9).

### Cost-Effectiveness Analysis

#### Base-Case Analysis

Imipenem/cilastatin, meropenem, ceftazidime/avibactam, and ceftolozane/tazobactam were included in the pharmacoeconomic analysis. The total costs and outcomes for the four treatment strategies were summarized in Table 2. Imipenem/cilastatin (the lowest cost in base-case analysis) was used as the baseline in calculating the ICERs of other strategies. The ICERs per QALY gained for meropenem, ceftazidime/avibactam, and ceftolozane/tazobactam relative to imipenem/cilastatin corresponded to US$579, US$24569, and US$29040, respectively.

**TABLE 2:** Cost-effectiveness of four strategies for cUTI patients due to *Enterobacteriaceae* infection.

Treatment strategy	Total cost (US$)	Incremental cost (US$)	QALY	QALY gained	ICER per QALY gained
Imipenem/cilastatin	9,150	—	4.500	—	—
Meropenem	9,209	59^a^	4.602	0.102^a^	579
Ceftazidime/avibactam	12,909	3759^a^	4.653	0.153^a^	24,569
Ceftolozane/tazobactam	12,025	2875^a^	4.599	0.099^a^	29,040

QALY, quality-adjusted life years; ICER, incremental cost-effectiveness ratio.

a Calculated as the average cost per patient and the average QALY gained per patient in this strategy minus those of the treatment of imipenem/cilastatin.

In the current study, ceftazidime/avibactam had the best efficacy with respect to QALY gained but it also was associated with the highest costs. Meropenem and ceftolozane/tazobactam were also associated with greater efficacy but higher costs than imipenem/cilastatin. Compared with ceftolozane/tazobactam, meropenem was a dominant option for cUTI treatment (i.e., it led to a higher QALY saved and was less costly). Among these four treatment strategies, meropenem treatment was preferred based on its exhibiting clinical efficacy at an acceptable cost. It was found that ceftazidime/avibactam and ceftolozane/tazobactam were not strongly recommended treatment strategies, since the ICERs per QALY gained corresponded to > US$10,121.3.

#### Sensitivity Analysis

Deterministic sensitivity analyses indicated that the clinical success of study drugs and daily drug costs had a high impact on the ICERs between the four treatment strategies. When the willingness-to-pay (WTP) threshold was set at US$10121.3, the clinical success of imipenem/cilastatin at higher than 84.7% (vs. meropenem), and the cost of ceftazidime/avibactam (vs. imipenem/cilastatin) and ceftolozane/tazobactam (vs. imipenem/cilastatin) at less than US$374 and US$281, respectively, would make the use of them become acceptable. Additionally, the clinical cure rate of imipenem/cilastatin at higher than 91 and 87.5%, respectively, would lead to evaluating it as superior to ceftazidime/avibactam and ceftolozane/tazobactam.

Results of probabilistic sensitivity analyses revealed that meropenem, ceftazidime/avibactam, and ceftolozane/tazobactam had probabilities of 69.3, 41.2, and 39.3%, respectively, of being cost-effective relative to imipenem/cilastatin under the threshold currently accepted in China (US$10,121.3) in patients with cUTI.

## Discussion

Our systematic review involved a total of 25 RCTs (24 included in the NMA), containing 15 antibiotics, and comprehensively evaluated the efficacy, safety and pharmacoeconomics of antibiotics used for *Enterobacteriaceae* infections. We integrated the existing evidence and found that 1) the SUCRA probabilities showed that the efficacy with the highest-ranking of interventions were meropenem/vaborbactam, meropenem, imipenem/cilastatin, ceftriaxone, ceftazidime/avibactam, and ceftolozane/tazobactam; 2) there were no significant differences in the safety outcomes of the several antibiotics mentioned above, but the incidence of adverse events of piperacillin/tazobactam was significantly lower than that of ceftazidime/avibactam (RR = 0.74) and imipenem/cilastatin (RR = 0.68); 3) in the patients with cUTI, ceftolozane/tazobactam and ceftazidime/avibactam had favorable clinical efficacy, but compared to imipenem/cilastatin, these two agents were not cost-effective strategies.

Some studies have been undertaken to compare the efficacy and safety of antibiotics used for *Enterobacteriaceae* infections (Che et al., 2019; Nguyen et al., 2019). However, to the best of our knowledge, our study is the first to comprehensively evaluate the efficacy, safety, and cost-effectiveness of antibiotics for the treatment of *Enterobacteriaceae* using NMA and decision-analytic Markov model. The use of NMA is of particular importance given the lack of direct comparative evidence for the various antibiotics and the inability to evaluate indirect evidence between RCTs. The twenty-five original studies we included are all RCTs which are recognized as the highest level of evidence, making our conclusions more credible than other meta-analyses that included both RCTs and observational studies (Nguyen et al., 2019). Then, a pharmacoeconomics analysis based on the results of NMA can better explore the clinical and economic benefits of these antibiotics for the infection caused by *Enterobacteriaceae* and to help better clinical decision-making.

CRE has become a major threat to public safety due to its high mortality and low clinical success rate, making treatment options for *Enterobacteriaceae* infections especially important. Vaborbactam is a novel *β*-lactamase inhibitor that exhibits excellent antibacterial efficacy when combined with meropenem. In our study, meropenem/vaborbactam had favorable efficacy for *Enterobacteriaceae* infections (overall ranking first). However, studies of this agent are limited because of the short time on the market. Only one study analyzing meropenem/vaborbactam was included in our NMA, resulting in a small number of populations. Hence, we were unable to obtain sufficient evidence to draw a robust conclusion on this agent. Notably, for ceftolozane/tazobactam, the efficacy was not optimal regardless of infection types (overall ranking sixth). However, in the cUTI subgroup, the overall ranking of ceftolozane/tazobactam rose to first place and its microbiological success was significantly higher than that of doripenem (RR = 1.13). Ceftolozane/tazobactam was more effective in cUTI, which may be related to the fact that the excretion ratio of ceftolozane and tazobactam in their original form is higher than other drugs (Yahav et al., 2020).

Ceftazidime/avibactam is the only one marketed in China among novel BL/BLIs. Our study found that ceftazidime/avibactam ranked fifth without distinguishing infection types, rose to second place followed ceftolozane/tazobactam in the cUTI subgroup. The reason for the difference in the efficacy of ceftazidime/avibactam may also due to its excretion ration through the kidneys *via* urine in their original form is higher than other drugs (Zhanel et al., 2013). Although the efficacy of ceftazidime/avibactam was favorable, the incidence of adverse events of ceftazidime/avibactam was higher than that of piperacillin/tazobactam (RR = 0.74). Previous studies also demonstrated that the incidence of serious adverse events of ceftazidime/avibactam was higher than that of comparators (e.g., carbapenems) (Sternbach et al., 2018; Che et al., 2019). Therefore, the safety of ceftazidime/avibactam needs to be further appraised. We found that ceftazidime/avibactam was more excellent on efficacy than ceftazidime alone, which may be due to avibactam can restore the activity of ceftazidime. In our study, the safety results of ceftazidime/avibactam and ceftazidime were similar, possibly because of the low potential for protein binding and drug-drug interactions (Merdjan et al., 2015).

In our research, meropenem had bettering efficacy and was likely to be cost-effective in the treatment of cUTI caused by *Enterobacteriaceae*. Nevertheless, the overuse of carbapenems has been noticed to be associated with the development of CRE. According to CHINET, the drug resistance rate of *Enterobacteriaceae* to carbapenems has increased from 3 to 11% in the last decade, especially imipenem and meropenem to *Klebsiella pneumoniae* raised from 3 to 26% in the last fifteen years (http://www.chinets.com/). Although the limited data on resistance in our study precluded us from performing any correlation analysis between studied agents and CRE development, previous studies have pointed out that increased exposure to carbapenems would lead to the occurrence of antimicrobial resistance (Kwak et al., 2005; Schwaber et al., 2008).

Our study found that neither ceftolozane/tazobactam nor ceftazidime/avibactam were cost-effective in patients with cUTI. This result can be explained by three possible reasons. First, we found that the resistance rate of ceftolozane/tazobactam to *Klebsiella pneumoniae* in China is 41.1%, which will affect the clinical success of treatment and increase the resistance-related cost. Second, ceftazidime/avibactam is less resistant to *Klebsiella pneumoniae* than that of imipenem/cilastatin, and the two agents have similar rates of resistance to *E. coli*. However, *E. coli* strains are the main pathogen of cUTI, so the superiority of ceftazidime/avibactam over drug-resistant strains could not be highlighted. Finally, the price of ceftolozane/tazobactam and ceftazidime/avibactam are both high. In a study analyzing extended-spectrum *β*-Lactamases-producing Gram-negative pathogens infections, ceftazidime/avibactam was not deemed cost-effective for patients with cUTI, consistent with our finding (Nguyen et al., 2019). However, a study published in Italy on imipenem and ceftazidime/avibactam in the treatment of cUTI found that ceftazidime/avibactam is cost-effective for cUTI patients (Kongnakorn et al., 2019a). The inconsistencies between the studies may be due to the large difference in the clinical success rate between imipenem and ceftazidime/avibactam, and the higher WTP threshold in Italy. The differences between the Italian research and our study are also consistent with the results of our sensitivity analyses, in which the clinical success rates and drug costs had a strong impact on the ICERs between the different treatment strategies. Our study only analyzed the cost-effectiveness of ceftazidime/avibactam in patients with cUTI, because antibiotics related to cIAI treatment cannot form a closed loop. Similarly, studies have been performed for bacteremia, cIAI, and hospital-acquired pneumonia/ventilator acquired pneumonia (Kongnakorn et al., 2019b; Simon et al., 2019; Tichy et al., 2020), and the pharmacoeconomics results of ceftazidime/avibactam were all favorable in these studies. The economic effects of ceftazidime/avibactam for different types of infection require more researches to confirm.

We need to admit that there are some limitations in our research: 1) some direct comparisons between drugs included few RCTs, resulting in wide CI for these drugs; 2) since most studies do not provide information on antibiotic resistance or enzyme production, it is impossible to evaluate the effects of antibiotics on *Enterobacteriaceae* with different drug resistance phenotypes; 3) the definitions of microbiological success and the incidence of adverse events in different studies are inconsistent; 4) we did not consider the cost related to adverse events in our cost-effectiveness analysis.

## Conclusion

In brief, our NMA indicated that the BL/BLIs (meropenem/vaborbactam, ceftolozane/tazobactam, and ceftazidime/avibactam) demonstrate favorable clinical efficacy in the treatment of infections caused by *Enterobacteriaceae*, however, ceftazidime/avibactam has a higher incidence of adverse events than that of piperacillin/tazobactam. For cost-effectiveness, only meropenem was the cost-effective strategy and neither ceftazidime/avibactam nor ceftolozane/tazobactam was recommended strategy in the treatment of patients with cUTI. Due to the limitation of the number of studies included, more clinical studies with a large sample and high-quality are needed to validate the findings of this study, further exploring the value of antimicrobials in *Enterobacteriaceae* infections and providing a scientific and rational basis for clinical work.

## Data Availability

The original contributions presented in the study are included in the article/Supplementary Material, further inquiries can be directed to the corresponding authors.

## Abbreviations

BL/BLIs, β-lactam/β-lactamase inhibitors; cIAI, complex intra-abdominal infection; CRE, carbapenem-resistant *Enterobacteriaceae*; cUTI, complex urinary tract infection; ICERs, incremental cost-effectiveness ratios; NMA, network meta-analysis; QALY, quality-adjusted life years; RCTs, randomized controlled trials; RR, relative risk; SUCRA, surface under the cumulative ranking curve; WTP, willingness-to-pay; 95% CI, 95% confidence interval.
