# Supplementary material for: Choosing Optimal Antibiotics for the Treatment of Patients Infected With Enterobacteriaceae: A Network Meta-analysis and Cost-Effectiveness Analysis
Source: Front Pharmacol. 2021 Jun 17;12:656790. doi: 10.3389/fphar.2021.656790 (PMC8245689; doi:10.3389/fphar.2021.656790)
Supplement: Supplementary file 1 [file DataSheet1.docx]

**Supplemental Material**

**Contents**

**Appendix 1** Search strategy of electronic databases

**Appendix 2** Details of methods used in cost-effectiveness analysis (Table S1 and Figure S1 - S5)

**Appendix 3** Main characteristics of the randomized controlled trials included in the meta-analysis (Table S2)

**Appendix 4** Risk of bias of included studies (Figure S6 and Figure S7)
**Appendix 5** Funnel plot of the meta-analysis for primary outcomes (Figure S8)

**Appendix 6** Results of complex urinary tract infection subgroup (Table S3 and Figure S9)

**Appendix 7** Results of secondary outcomes (Figure S10 - S12)

**Appendix 8** Results of sensitivity analysis with a sample size more than 30 (Table S4 and Figure S13 - S15)

**Appendix 9** Results of sensitivity analysis with a sample size more than 100 (Table S5 and Figure S16)

**Appendix 1**

**Search strategy of electronic databases (PubMed as sample):**

#1 Enterobacteriaceae [MeSH Terms] OR Enterobacteriaceae [Text Word]

#2 Enterobacter [MeSH Terms] OR Enterobacter [Text Word]

# #3 Carbapenem-Resistant Enterobacteriaceae [MeSH Terms] OR Carbapenem-Resistant Enterobacteriaceae [Text Word]

#4 Citrobacter [MeSH Terms] OR Citrobacter [Text Word]

#5 Cronobacter [MeSH Terms] OR Cronobacter [Text Word]

#6 Klebsiella [MeSH Terms] OR Klebsiella [Text Word]

#7 Proteus [MeSH Terms] OR Proteus [Text Word]

#8 Serratia [MeSH Terms] OR Serratia [Text Word]

#9 Escherichia [MeSH Terms] OR Escherichia [Text Word]

#10 gram-negative bacilli [Text Word]

#11 OR/#1-10

#12 meropenem [MeSH Terms] OR meropenem [Text Word]

#13 vaborbactam [Supplementary Concept] OR vaborbactam [Text Word]

#14 doripenem [MeSH Terms] OR doripenem [Text Word]

#15 ertapenem [MeSH Terms] OR ertapenem [Text Word]

#16 cefepime [MeSH Terms] OR cefepime [Text Word]

#17 cefotaxime [MeSH Terms] OR cefotaxime [Text Word]

#18 ceftriaxone [MeSH Terms] OR ceftriaxone [Text Word]

#19 ciprofloxacin [MeSH Terms] OR ciprofloxacin [Text Word]

#20 levofloxacin [MeSH Terms] OR levofloxacin [Text Word]

#21 fosfomycin [MeSH Terms] OR fosfomycin [Text Word]

#22 tigecycline [MeSH Terms] OR tigecycline [Text Word]

#23 minocycline [MeSH Terms] OR minocycline [Text Word]

#24 polymyxin [MeSH Terms] OR polymyxin [Text Word]

#25 gentamicin [MeSH Terms] OR gentamicin [Text Word]

#26 amikacin [MeSH Terms] OR amikacin [Text Word]

#27 imipenem [MeSH Terms] OR imipenem [Text Word]

#28 cilastatin [MeSH Terms] OR cilastatin [Text Word]

#29 ceftolozane [Supplementary Concept] OR ceftolozane [Text Word]

#30 tazobactam [MeSH Terms] OR tazobactam [Text Word]

#31 ceftazidime [MeSH Terms] OR ceftazidime [Text Word]

#32 avibactam [Supplementary Concept] OR avibactam [Text Word]

#33 piperacillin [MeSH Terms] OR piperacillin [Text Word]

#34 cefoperazone [Supplementary Concept] OR cefoperazone [Text Word]

#35 sulbactam [Supplementary Concept] OR sulbactam [Text Word]

#36 carbapenems [MeSH Terms] OR carbapenems [Text Word]

#37 OR /#12-36

#38 random*[Text Word]

#39 (Randomized Controlled trial [Publication Type]) OR (Randomized Controlled trials as topic [MeSH Terms]) OR (Controlled Clinical trial [Publication Type])

#40 #38 OR #39

#41 #11 AND #37 AND #40

**Appendix 2**

**Details of methods used in cost-effectiveness analysis**

*Model structure*

Given that the best available evidence for patients with complex urinary tract infection (cUTI) (more close-loop formed), we only chose this infection type for economic evaluation.

A combined decision analytical Markov model was constructed to estimate the cost-effectiveness of initiating empirical antibiotics treatment for cUTI patients caused by *Enterobacteriaceae*, using the software of TreeAge Pro 2011 (TreeAge Software, Inc., MA, USA). Patients entered the model at the time of cUTI diagnosis, and if microbiological results become available and showed the presence of *Enterobacteriaceae*. The model then generated several identical cohorts for receiving different antibiotics treatment.

As shown in Figure S1, the patient continued empirical treatment until microbiological results were available (2–3 days). The patient was considered as a clinical failure if microbiological results revealed that at least one of the pathogens was resistant to empirical treatment and may then switched to the next treatment line (i.e., salvage therapy with a combination of colistin and high dose carbapenem) or die from infection. If no resistance was observed, empirical treatment was continued as definitive therapy. Once the patient completed the treatment course, the patient was assessed to evaluate for response at the end-of-treatment assessment. If there was no response, the patient was counted as having a failure and switched to the salvage therapy or die from infection. Patients who received salvage therapy might have clinical cure or still die due to the infection. Patients with clinical cure entered a 5-year Markov model. Three states in the Markov model are: 1, recurrence of infection (i.e., equivalent to clinical failure observed at the long-term follow-up visit in the randomized controlled studies); 2, infection-free survival; and 3, death.

*Study drugs*

In patients with cUTI, antibiotics with a high surface under the cumulative ranking curve probabilities in network meta-analysis and marketed in China were selected for economic evaluation.

*Model inputs and data resources*

The key input parameters were summarized in the Table S1. The epidemiological data (distribution of *Enterobacteriaceae* and resistance rate of study drugs) were derived from the CHINET 2019 (<http://www.chinets.com/>). The rate of clinical success was derived from the systematic review and meta-analysis of this study.

The Chinese health-care perspective was adopted in the economic modelling. Hospital costs per day and cost by resistant pathogen were obtained from a published paper from China([1](#_ENREF_1)). The costs of drug acquisition were obtained from the China Pharmaceutical Procurement Platform (<https://yp.bjmbc.org.cn/view/index/guide.html>). All costs were recorded in Chinese yuan and then converted into US dollars (exchange rate: 1 yuan = US$0.1521). Health utilities were based on data obtained from published literature([2](#_ENREF_2), [3](#_ENREF_3)).

*Outcomes and Sensitivity analyses*

We set the time horizon of the model as five years to cover the infection episode and to evaluate long-term impact. The model employed an annual discount rate of 3% on costs and health benefits.

The outcomes of interest were total costs and quality-adjusted life years (QALY) gained. The incremental cost-effectiveness ratio (ICER) per additional QALY gained was calculated, which was used to compare the performance of treatment strategies. We considered treatment strategies with an ICER of less than US$10121.3 (i.e., Chinese gross domestic product per capita in 2019) per QALY gained to be acceptable.

Deterministic sensitivity analyses were used to investigate the potential influence of variations of key variables on the ICERs and determine which variables were most sensitive to the results. Monte Carlo probabilistic sensitivity analysis was carried out with 1,000 times of Monte Carlo simulations to evaluate the impact of all variables simultaneously. The variation ranges of the key variables are listed in Table S1.

**Figure S1** Decision analysis and Markov combination model. R: Recurrence; S: Survival; D: Death.


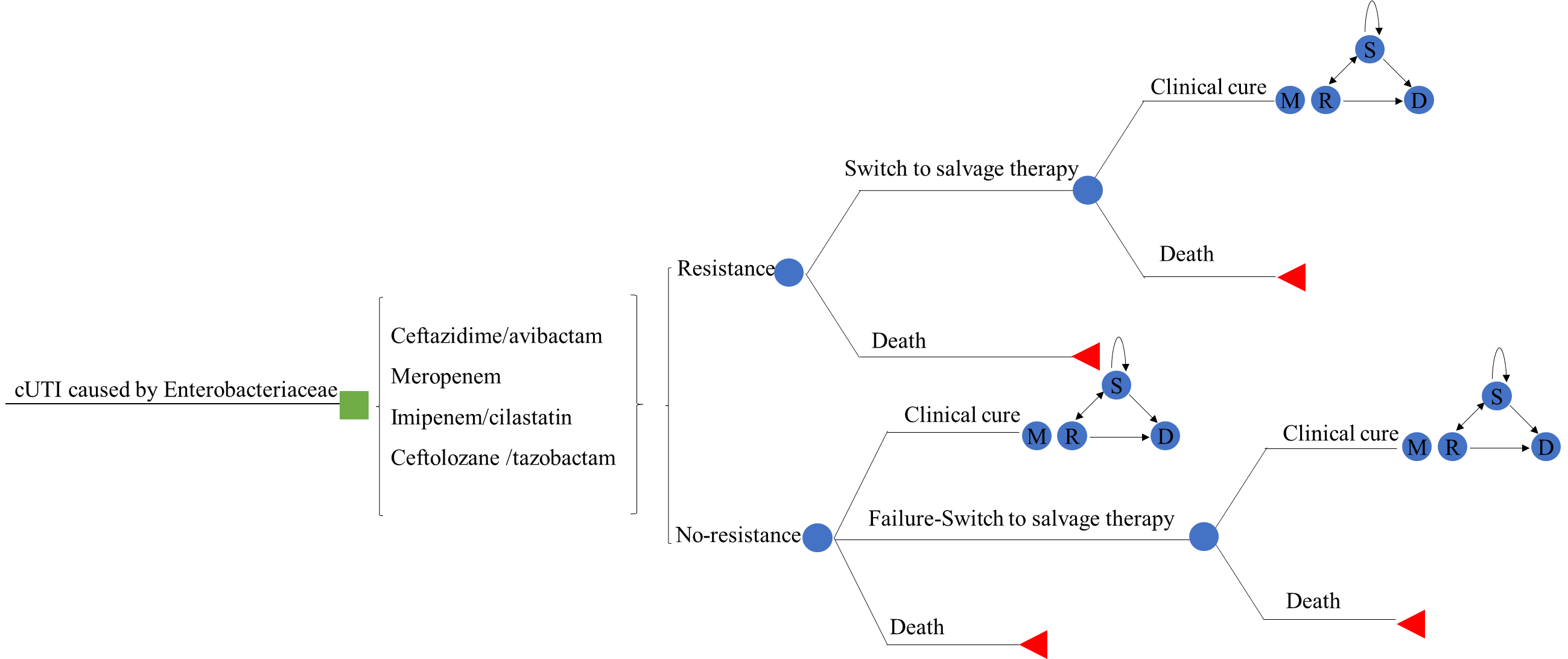


**Table S1. Parameter in Base-case and sensitivity analyses used in cost-effectiveness**

| **Parameter** | **Base case** | **Sensitivity Range** | **References** |
| --- | --- | --- | --- |
| **Epidemiology inputs** |  |  | CHINET 2019 |
| Distribution of Enterobacteriaceae from urine culture |  |  |  |
| *E. coli* | 77.2% |  |  |
| *K. pneumoniae* | 16.0% |  |  |
| *Other Gram-negative pathogens* | 6.8% |  |  |
| Resistance rates |  |  |  |
| Ceftazidime/avibactam |  |  |  |
| *E. coli* | 4.5% |  |  |
| *K. pneumoniae* | 5.4% |  |  |
| *Other Gram-negative pathogens* | 8.2% |  |  |
| Meropenem |  |  |  |
| *E. coli* | 2.1% |  |  |
| *K. pneumoniae* | 25.9% |  |  |
| *Other Gram-negative pathogens* | 11.4% |  |  |
| Imipenem/cilastatin |  |  |  |
| *E. coli* | 2.0% |  |  |
| *K. pneumoniae* | 24.5% |  |  |
| *Other Gram-negative pathogens* | 11.5% |  |  |
| Ceftolozane/tazobactam |  |  |  |
| *E. coli* | 10.6% |  |  |
| *K. pneumoniae* | 41.1% |  |  |
| *Other Gram-negative pathogens* | 25.1% |  |  |
| **Clinical inputs** |  |  |  |
| Duration of definitive therapy (days) | 7 | 5–14 (Triangular) | ([4](#_ENREF_4), [5](#_ENREF_5)) |
| Length of stay (days) |  |  |  |
| With clinical success | 10.4 | 7-15 (Triangular) | ([6](#_ENREF_6)) |
| Without clinical success | 14.2 | 10-18 (Triangular) | ([6](#_ENREF_6)) |
| Duration to obtain microbial results (days) | 3 | 2–3 (Uniform) | ([4](#_ENREF_4), [5](#_ENREF_5)) |
| Clinical success in cUTI |  |  |  |
| Imipenem/cilastatin | 0.81 | 0.68–0.93(Lognormal) | Pooled data |
| Meropenem^1^ | 0.89 | 0.83–0.94 (Lognormal) | Pooled data |
| Ceftolozane/tazobactam | 0.92 | 0.89–0.95 (Lognormal) | Pooled data |
| Ceftazidime/avibactam | 0.90 | 0.87–0.93(Lognormal) | Pooled data |
| Colistin + high-dose carbapenem^2^ | 0.904 | 0.88–0.93 (Lognormal) | ([6](#_ENREF_6)) |
| Probability of in-hospital death |  |  |  |
| Appropriate empirical treatment | 0.018 | 0.009–0.027 (Beta) | ([7](#_ENREF_7)) |
| Inappropriate empirical treatment | 0.072 | 0.036–0.108 (Beta) | ([7](#_ENREF_7)) |
| Resistant to empirical treatment | 0.0864^3^ | 0.0432–0.1296 (Beta) | ([7](#_ENREF_7)) |
| Mortality rate when treated with salvage therapy | 0.20 | 0.10–0.30 (Uniform) | ([8](#_ENREF_8)) |
| Probability of recurrence |  |  |  |
| Meropenem^2^ | 0.085 |  | ([6](#_ENREF_6)) |
| Ceftazidime/avibactam | 0.0704 |  | ([6](#_ENREF_6)) |
| Ceftolozane/tazobactam | 0.036 |  | ([9](#_ENREF_9)) |
| Imipenem/cilastatin | 0.067 |  | ([10](#_ENREF_10)) |
| Colistin + high-dose carbapenem^2^ | 0.085 |  | ([6](#_ENREF_6)) |
| **Economic inputs** |  |  |  |
| Antibiotic cost per day ($) |  |  | China Pharmaceutical Procurement Platform |
| Meropenem | 140.2 | 70.1–210.3 (Gamma) |  |
| Ceftazidime/avibactam | 627.3 | 313.7–941.0 (Gamma) |  |
| Ceftolozane/tazobactam^4^ | 535.8 | 267.9–803.7 (Gamma) |  |
| Imipenem/cilastatin | 80.9 | 40.5–121.4 (Gamma) |  |
| Colistin | 1035.0 | 517.5–1552.5(Gamma) |  |
| Cost by resistant pathogen ($) | 2311 | 1115.5–3466.5 (Gamma) | ([1](#_ENREF_1)) |
| Hospital cost per day ($) | 411.9 | 206.0–617.9 (Gamma) | ([1](#_ENREF_1)) |
| **Utility (quality of life)** |  |  |  |
| With clinical cure | 0.92 |  | ([2](#_ENREF_2)) |
| Without clinical cure | 0.61 |  | ([3](#_ENREF_3)) |

cUTI: complex urinary tract infection.

^1^ The clinical cure of meropenem was derived from complex intra-abdominal infection studies due to the lack of complex urinary tract infection data.

^2^ Assumed to be the same as doripenem in RECAPTURE trial, given the same drug class.

^3^ Assumed to be 20% higher than mortality among patients with susceptible pathogens but had inappropriate empirical therapy.

^4^ This price is the estimated price in consultation with Merck Medical Department (assuming the price is 85% of the price of ceftazidime-avibactam, based on the drug price in the Netherlands([8](#_ENREF_8))).

**Figure S2** Clinical success of imipenem/cilastatin.

**Figure S3** Clinical success of meropenem.

**Figure S4** Clinical success of ceftolozane/tazobactam.

**Figure S5** Clinical success of ceftazidime/avibactam.

**Appendix 3**

**Table S2** Main characteristics of the randomized controlled trials (RCTs) included in the meta-analysis

|  | Design | Country | Type of infection | Organism | Antibiotic group | Sample size | Mean age | Median  duration | Dosage regimen | Clinical cure (n/N)^1^ | Microbiological cure (n/N)^1^ | AE (n/N)^1^ | Mortality (n/N)^1^ | Industry Sponsorship |
| --- | --- | --- | --- | --- | --- | --- | --- | --- | --- | --- | --- | --- | --- | --- |
| Kaye2019 ([11](#_ENREF_11)) | multicenter  randomized double-blind Phase 2/3 trial | 16 countries^2^ | cUTI/AP | E (91.8%) | FOS | 233 | 49.9 | 7.1 | i.v. 6 g q8 h | 167/184 | 121/184 | 98/233 | 0/233 | Zavante Therapeutics |
|  |  |  |  |  | PT | 231 | 51.3 | 7.1 | i.v. 4.5 g q8 h | 163/178 | 100/178 | 74/231 | 0/231 |  |
| Patrick 2018([12](#_ENREF_12)) | multicenter  randomized open-label | 9 countries^3^ | cUTI/AP | E (100%) | PT | 188 | 70 | NR | i.v. 4.5 g q6 h | NR | 169/174 | NR | 23/187 | University of Queensland |
|  |  |  |  |  | MEPM | 191 | 69 |  | i.v. 1 g q8 h |  | 184/185 |  | 7/191 |  |
| Seo 2017 ([13](#_ENREF_13)) | multicenter  randomized open-label | Korea | UTI | E (100%) | PT | 33 | 68.8 | NR | Ccr > 40 mL/min 4.5 g q6 h, Ccr of 20-40 mL/min 2.25 g q6 h, Ccr < 20 mL/min 8 g q8 h | 31/33 | 32/33 | NR | 2/33 | Korean Health Technology R&D Project |
|  |  |  |  |  | CFPM | 6 | 75.3 |  | Ccr > 60 mL/min 2 g q12 h, Ccr of 30-60 mL/min 2 g q24 h, Ccr < 30 mL/min 1 g q24 h | 2/6 | 2/6 |  | 2/6 |  |
|  |  |  |  |  | ETPM | 33 | 65.2 |  | Ccr > 30 mL/min 1 g q24 h, Ccr ≤ 30 mL/min 500 mg daily | 32/33 | 32/33 |  | 2/33 |  |
| Xinyu qin  2017([14](#_ENREF_14)) | multicenter randomized double-blind Phase 3 | China | cIAI | E (86.6%) | CA | 215 | 48.5 | NR | i.v. 2 g/0.5 g q8 h | 166/177 | 92/99 | 82/215 | 2/215 | AstraZeneca and Pfizer |
|  |  |  |  |  | MEPM | 217 | 48.5 |  | i.v. 1 g q8 h | 173/184 | 107/113 | 83/217 | 1/217 |  |
| Wagenlehner 2016([6](#_ENREF_6)) | multicenter  randomized double-blind Phase 3 | 25 countries^4^ | cUTI | E (95.2%) | CA | 393 | 51.4 | 7 | i.v. 2 g/0.5 g q8 h | 355/393 | 304/393 | 185/511 | 0/511 | AstraZeneca and Actavis |
|  |  |  |  |  | DOPM | 417 | 53.3 | 8 | i.v. 0.5 g q8 h | 377/417 | 296/417 | 158/509 | 0/509 |  |
| Mazuski 2016([15](#_ENREF_15)) | multicenter randomized double-blind | 30 countries^5^ | cIAI | E (87%) | CA | 529 | 49.8 | 8 | i.v. 2 g/0.5 g q8 h | 429/520 | 337/413 | 243 /529 | 13/529 | AstraZeneca |
|  |  |  |  |  | MEPM | 529 | 50.3 | 8.3 | i.v. 1 g q8 h | 444/523 | 349/410 | 227/529 | 8/529 |  |
| Wagenlehner 2015([9](#_ENREF_9)) | multicenter randomized double-blind | 25 countries^6^ | cUTI | E (92%) | CT | 534 | 49.1 | NR | i.v. 1.5 g q8 h | 366/398 | 320/398 | 185/533 | 1/533 | Cubist Pharmaceuticals |
|  |  |  |  |  | LEFC | 534 | 48·1 |  | i.v. 0.75 g q.d. | 356/402 | 290/402 | 184/535 | 0/535 |  |
| Solomkin 2015([16](#_ENREF_16)) | multicenter randomized double-blind | 196 study centers worldwide^2^ | cIAI | E (91.1%) | CT | 487 | 50.8 | NR | i.v. 1.5 g q8 h | 323/389 | 259/275 | 212/482 | 11/482 | Cubist Pharmaceuticals |
|  |  |  |  |  | MEPM | 506 | 50.84 |  | i.v. 1 g q8 h | 364/417 | 304/321 | 212/497 | 8/497 |  |
| Vazquez 2012([10](#_ENREF_10)) | multicenter randomized patient-blinded Phase 2 | Guatemala, India, Jordan, Lebanon and America | cUTI | E (96.7%) | CA | 68 | 46.4 | 5 | i.v. 0.5 g/0.125 g q8 h | 24/28 | 19/27 | 46/68 | 0/68 | Novexel |
|  |  |  |  |  | IC | 67 | 48.2 | 6 | i.v. 0.5 g q6 h | 29/36 | 25/35 | 51/67 | 0/67 |  |
| Park 2012 ([17](#_ENREF_17)) | multicenter  randomized  double-blinded | Korea | cUTI | E (96.3%) | ETPM | 135 | 55.4 | NR | i.v. 1g q.d. | NR | 58/66 | 14/132 | NR | NR |
|  |  |  |  |  | CRO | 136 | 56.5 |  | i.v. 2 g q.d. |  | 63/71 | 6/135 |  |  |
| Nurgul 2010([18](#_ENREF_18)) | randomized single-blind | Turkey | UTI | E (96.7%) | FOS | 77 | 34 | NR | 3 g q.d. | 64/77 | 64/77 | 3/77 | NR | NR |
|  |  |  |  |  | CIP | 65 | 31 |  | 0.5 g q12h | 53/65 | 51/65 | 2/65 |  |  |
| Howard A 2007([19](#_ENREF_19)) | multicenter  randomized  double-blind | America | AP | E (92.7%) | LEFC | 146 | 38.9 | NR | i.v. or p.o. 0.75 g | 80/94 | 78/94 | 64/146 | 0/146 | PriCara |
|  |  |  |  |  | CIP | 165 | 39.4 |  | i.v. 0.4 g or p.o. 0.5 g bid. | 79/98 | 77/98 | 65/166 | 0/166 |  |
| Wilbur G 2004([20](#_ENREF_20)) | randomized double-blind | America | cUTI | E (94.2%), | ETPM | 473 | 51.8 | 4 | i.v. 1g q.d. | 232/256 | 229/256 | 129/468 | NR | Merck & Co., Inc |
|  |  |  |  |  | CRO | 377 | 52.6 | 4 | i.v. 1 g q.d. | 207/224 | 204/224 | 113/372 |  |  |
| Alfredo 1999([21](#_ENREF_21)) | randomized  open-label | America | Mixed infection types | E (80.5%) | CFPM | 25 | 49.5 | NR | i.v. 1 g b.i.d | 19 /22 | 15/18 | 2/25 | NR | Bristol–Myers Squibb Co |
|  |  |  |  |  | CAZ | 25 | 54.4 |  | i.v. 1 g t.i.d. | 17/22 | 9/14 | 0/25 |  |  |
| Shan-Chwen Chang 1998 ([22](#_ENREF_22)) | randomized open-label | China | Mixed infection types | E (85%) | CFPM | 21 | NR | NR | i.v. 2 g q12 h | 19/20 | 8/9 | NR | NR | NR |
|  |  |  |  |  | CAZ | 19 |  |  | i.v. 2 g q8 h | 15/16 | 6/7 |  |  |  |
| JOSESIFUENTES 1989([23](#_ENREF_23)) | randomized | America | Mixed infection types | E (82%) | CIP | 38 | 23 | NR | i.v. 0.2 g q8 h | 29/33 | 28/33 | 6/33 | 3/33 | NR |
|  |  |  |  |  | CAZ | 32 | 13 |  | i.v. 1 g q8 h | 19/26 | 22/26 | 3/26 | 2/26 |  |
| GEORGE A 1998([24](#_ENREF_24)) | multicenter randomized  double-blind | America and Canada | AP | E (84%) | LEFC | 89 | 41 | NR | p.o. 0.25 g q.d. | 82/89 | NA | 3/124 | 0/124 | NR |
|  |  |  |  |  | CIP | 58 | 34 |  | p.o. 0.5 g b.i.d | 51/58 |  | 6/80 | 0/80 |  |
| FERNANDO 2002([25](#_ENREF_25)) | Multicenter  randomized double-blind | America | cUTI | E (84.2%) | ETPM | 175 | 52.7 | NR | i.v. 1 g q.d. | NR | 83/97 | 21/175 | 0/175 | NR |
|  |  |  |  |  | CRO | 83 | 52.1 |  | i.v. 1g q.d. |  | 45/53 | 5/83 | 0/83 |  |
| Erasmo 2004([26](#_ENREF_26)) | multicenter randomized open-label  Phase 4 | China, Hong Kong, Malaysia, Korea, Philippines, and Thailand | cIAI | E (83.6%) | PT | 149 | 42.9 | NR | i.v. 4 g/0.5 g q8 h | 124/149 | 74/87 | 40/149 | 3/149 | Wyeth Pharmaceuticals, Inc |
|  |  |  |  |  | IC | 144 | 41.3 |  | i.v. 0.5 g/0.5 g q6 h | 126/144 | 76/83 | 52/144 | 0/144 |  |
| LAUREL 1995([27](#_ENREF_27)) | multicenter  randomized double-blind | America | UTI | E (84%) | CFPM | 26 | 60.6 | NR | i.v. 0.5 g q12 h | 22/26 | 22/26 | 6/39 | 0/39 | Bristol-MyersSquibb, Princeton,New Jersey. |
|  |  |  |  |  | CAZ | 22 | 63.4 |  | i.v.0.5 g q12 h | 20/22 | 20/22 | 4/38 | 0/38 |  |
| Kevin 2002 ([28](#_ENREF_28)) | multicenter randomized  double-blind | America and Europe | pyelonephritis, or other CUTI without acute pyelonephritis | E (88.7%) | ETPM | 298 | 51.3 | NR | i.v. 1 g q.d. | NR | 146/159 | 27/278 | 0/278 | Merck & Co., Inc. |
|  |  |  |  |  | CRO | 294 | 53 |  | i.v. 1 g q.d. |  | 159/171 | 20/278 | 0/278 |  |
| Kaye 2018 ([29](#_ENREF_29)) | multicenter randomized double-blind Phase 3 | 17 countries^7^ | cUTI, AP | E (89.1%) | MV | 274 | 53 | NR | i.v. 2 g/2 g q8 h | 174/192 | 128/192 | 106/272 | 2/272 | The Medicines Company and the Department of Health and Human Services |
|  |  |  |  |  | PT | 276 | 52.6 |  | i.v. q8 h 4 g/0.5 g | 157/182 | 105/182 | 97/273 | 2/273 |  |
| FOMIN 2008([30](#_ENREF_30)) | randomized double-blind Phase 3 | multicenter^2^ | cUTI | E (87.1%), | TGC | 301 | NR | NR | i.v. 0.1 g q12 h | 247/283 | 219/237 | 198/347 | NR | Wyeth Research |
|  |  |  |  |  | IC | 306 |  |  | i.v. 0.5 g/0.5 g q6 h | 228/237 | 198/223 | 198/355 |  |  |
| Naber 2009 ([31](#_ENREF_31)) | multicenter randomized double-blind Phase 3 | America, and Europe | cUTI pyelonephritis | E (90.9%) | DOPM | 377 | 51.2 | NR | i.v. 0.5 g q12 h | 272/286 | 230/280 | 240/376 | 0/376 | Johnson & Johnson |
|  |  |  |  |  | LEFC | 376 | 51.1 |  | i.v. 0.25 g q.d. | 240/266 | 221/265 | 222/372 | 0/372 |  |
| Carmeli 2016([32](#_ENREF_32)) | international randomized  open-label phase 3 | 16 countries^2^ | cUTIs | E (93.5%) | CA | 144 | 64.3 | 10 | i.v. 0.5 g /0.125 g q8 h | 132/144 | 118/144 | 43/152 | 3/152 | AstraZeneca and Forest Laboratories |
|  |  |  |  |  | CAR | 137 | 61.3 | 10 | NR | 129/137 | 88/137 | 54/153 | 3/153 |  |
|  |  |  | cIAI | E (95.2%) | CA | 10 | 49.9 | 10.5 | i.v. 0.5 g /0.125 g q8 h | 8/10 | 8/10 | 8/12 | 0/12 |  |
|  |  |  |  |  | CAR | 11 | 68.4 | 12 | NR | 6/11 | 6/11 | 12/15 | 1/15 |  |

CA: ceftazidime/avibactam, MV: meropenem/vaborbactam, MEPM: meropenem, DOPM: doripenem, IC: imipenem/cilastatin, CAZ: ceftazidime, CFPM: cefepime, CIP: ciprofloxacin, PT: piperacillin/tazobactam, ETPM: ertapenem, LEFC: levofloxacin, FOS: fosfomycin, CT: ceftolozane/tazobactam, TGC: tigecycline, CRO: ceftriaxone, CAR: carbapenems.

E: *Enterobacteriaceae,* NR: not report, AE: adverse events, cUTI: complex urinary tract infection, cIAI: Complicated intra-abdominal infection, AP: acute pyelonephritis, UTI: urinary tract infection.

^1^The data was shown as the events divided by the total numbers.

^2^The specific countries are not shown in detail in the original study.

^3^Australia, New Zealand, Singapore, Italy, Turkey, Lebanon, South Africa, Saudi Arabia and Canada.

^4^Eastern Europe, North America and Western Europe, Argentina, Brazil, Israel, Japan, Korea, Mexico, Peru and Taiwan.

^5^Argentina, Belgium, Brazil, Bulgaria, Canada, Chile, Croatia, Czech Republic, France, Germany, Greece, Hungary, India, Israel, Italy, Latvia, Lithuania, Malaysia, Mexico, Netherlands, Peru, Romania, Russian Federation, South Africa, Spain, Taiwan and the province of China, Thailand, Turkey, Ukraine and America.

^6^Bulgaria, Croatia, Estonia, Georgia, Hungary, Latvia, Moldova, Poland, Romania, Russia, Serbia, Slovakia, Slovenia, Ukraine, Mexico, USA, Brazil, Chile, Colombia, Peru, India, Israel, South Africa, South Korea and Thailand.

^7^Belarus, Brazil, Bulgaria, Czech Republic, Greece, Hungary, Italy, Peru, Poland, Romania, Slovakia, Slovenia, South Korea, Spain, Taiwan, Ukraine and America.

**Appendix 4**

**Figure S6** Risk of bias graph.


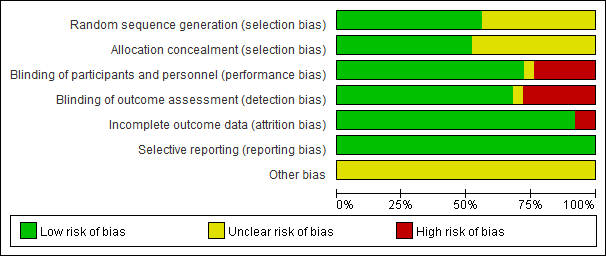


**Figure S7** Risk of bias summary.


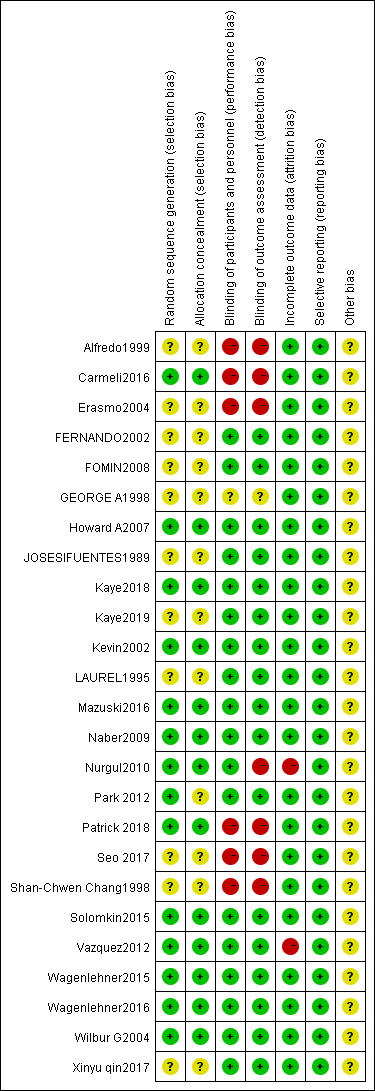


**Appendix 5**

**Figure S8** Funnel plot of the meta-analysis for primary outcomes. Figure A: clinical success, Figure B: microbiological success. A: ceftazidime/avibactam, B: meropenem/vaborbactam, C: meropenem, D: doripenem, E: imipenem/cilastatin, F: ceftazidime, G: cefepime, H: ciprofloxacin, I: piperacillin/tazobactam, G: ertapenem, K: levofloxacin, L: fosfomycin, M: ceftolozane/tazobactam, N: tigecycline, O: ceftriaxone.


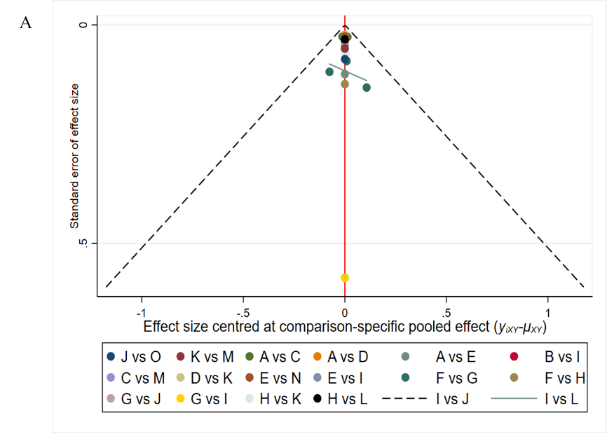

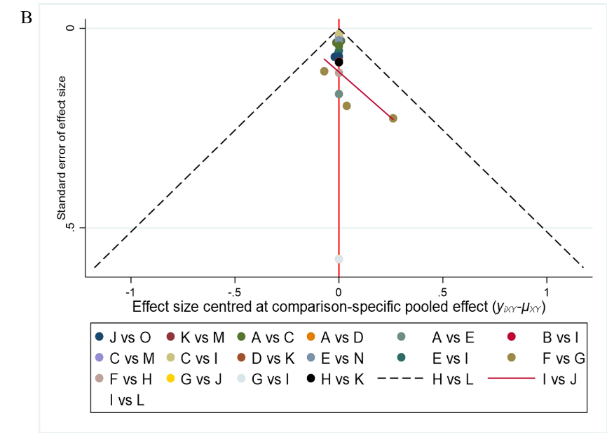


**Appendix 6**

**Table S3** Surface under the cumulative ranking curve (SUCRA) ranking list for complex urinary tract infection subgroup

| Treatment | Clinical success | |  | Microbiological success | | Overall Rank^1^ |
| --- | --- | --- | --- | --- | --- | --- |
|  | SUCRA | Rank |  | SUCRA | Rank |  |
| Ceftolozane/tazobactam | 71 | 3 |  | 86.3 | 1 | 1 |
| Ceftazidime/avibactam | 76.9 | 2 |  | 78.2 | 3 | 2 |
| Doripenem | 78.1 | 1 |  | 54.6 | 8 | 3 |
| Meropenem/vaborbactam | 68.2 | 5 |  | 64.1 | 6 | 4 |
| Imipenem/cilastatin | 56.1 | 7 |  | 70.3 | 4 | 5 |
| Tigecycline | 30 | 12 |  | 80.6 | 2 | 6 |
| Levofloxacin | 52.1 | 8 |  | 60.6 | 7 | 7 |
| Fosfomycin | 43 | 10 |  | 66 | 5 | 8 |
| Ceftriaxone | 68.2 | 4 |  | 34.8 | 11 | 9 |
| Ertapenem | 59.1 | 6 |  | 28.5 | 12 | 10 |
| Ciprofloxacin | 36.1 | 11 |  | 48 | 9 | 11 |
| Piperacillin/tazobactam | 46.3 | 9 |  | 26.9 | 13 | 12 |
| Ceftazidime | 10.1 | 13 |  | 8.4 | 14 | 13 |
| Cefepime | 5 | 14 |  | 4.1 | 15 | 14 |
| Meropenem | - | - |  | 38.4 | 10 | - |

The orders were from best to worst.

^1^adding the order of clinical success and microbiological success together (Interventions with the same ranking would be re-ranked based on the sum of their SUCRA probabilities).

**Figure S9** Clinical success and microbiological success of interventions for complex urinary tract infection subgroup. Antibiotics were sorted in the order of decreasing clinical success. The clinical success results were compared from left to right, and the microbiological success results should be read from right to left (the results were expressed by relative risk with 95% confidence interval). Significant results were shown in bold and underlined. DOPM: doripenem, CA: ceftazidime/avibactam, CT: ceftolozane/tazobactam, CRO: ceftriaxone. MV: meropenem/vaborbactam, ETPM: ertapenem, IC: imipenem/cilastatin, LEFC: levofloxacin, PT: piperacillin/tazobactam, FOS: fosfomycin, CIP: ciprofloxacin, TGC: tigecycline, CAZ: ceftazidime, CFPM: cefepime, MEPM: meropenem.


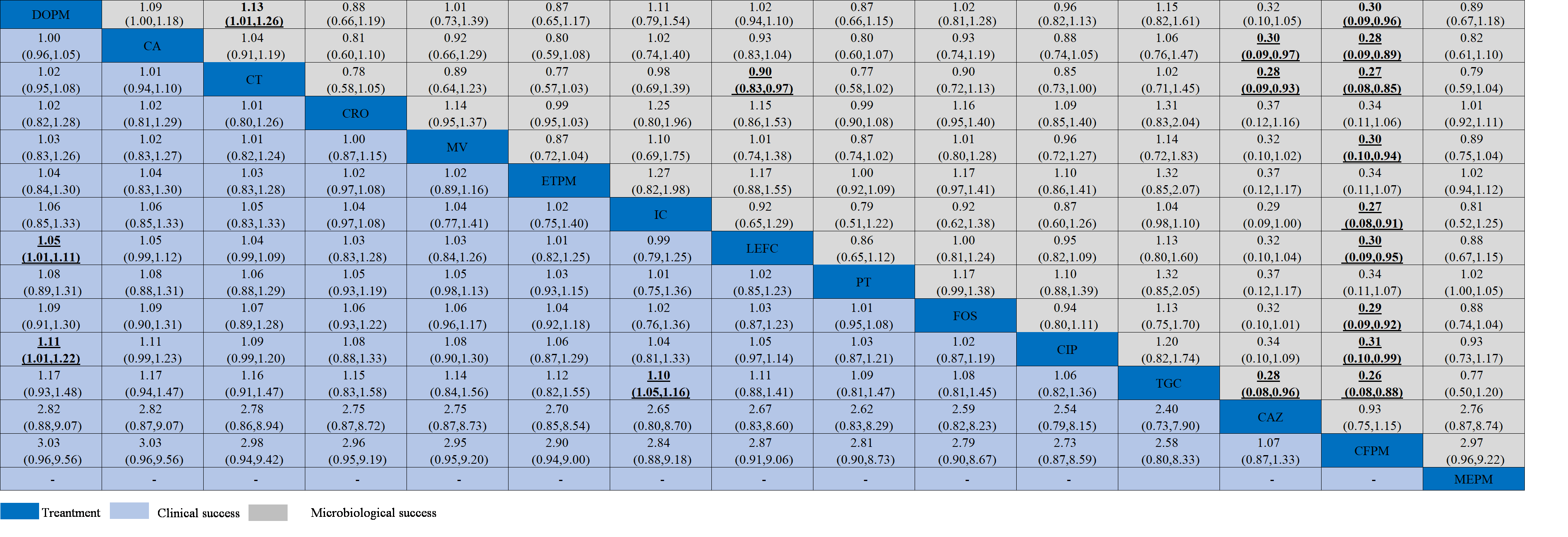


**Appendix 7**

**Figure S10** Forest plots showing relative risk with 95% confidence interval of adverse events in a random-effects model. cUTI: complex urinary tract infection; cIAI: complicated intra-abdominal infection; CA: ceftazidime/avibactam; CAR: carbapenems.


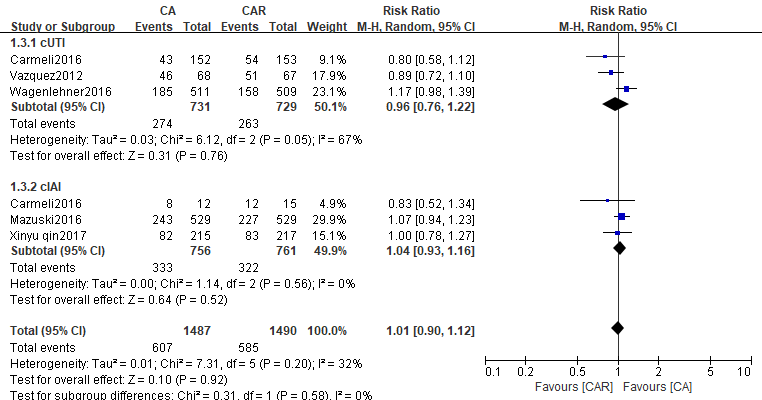


**Figure S11** Forest plots showing relative risk with 95% confidence interval of mortality in a random-effects model. cUTI: complex urinary tract infection; cIAI: complicated intra-abdominal infection; CA: ceftazidime/avibactam; CAR: carbapenems.


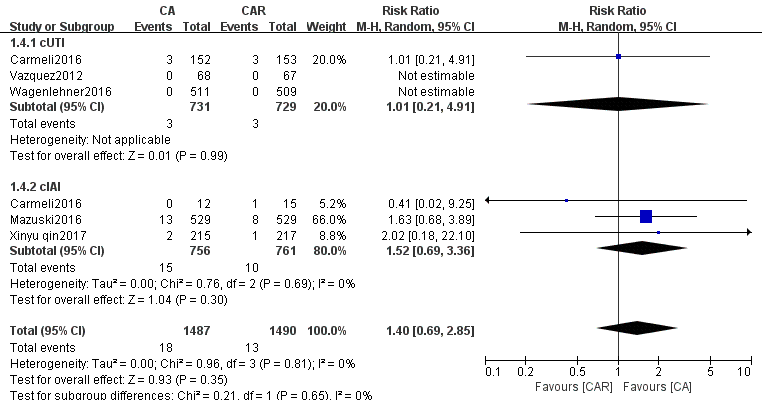


**Figure S12** Adverse events and mortality of interventions in the treatment of *Enterobacteriaceae* infections. Drugs were sorted in the order of increasing mortality. The adverse events results were compared from left to right, and the mortality results should be read from right to left (the results were expressed by relative risk with 95% confidence interval). Significant results were shown in bold and underlined. MEPM: meropenem, IC: imipenem/cilastatin, LEFC: levofloxacin, CT: ceftolozane/tazobactam, CA: ceftazidime/avibactam, DOPM: doripenem, ETPM: ertapenem, FOS: fosfomycin, CIP: ciprofloxacin, CAZ: ceftazidime, MV: meropenem/vaborbactam, CRO: ceftriaxone, PT: piperacillin/tazobactam, CFPM: cefepime, TGC: tigecycline.


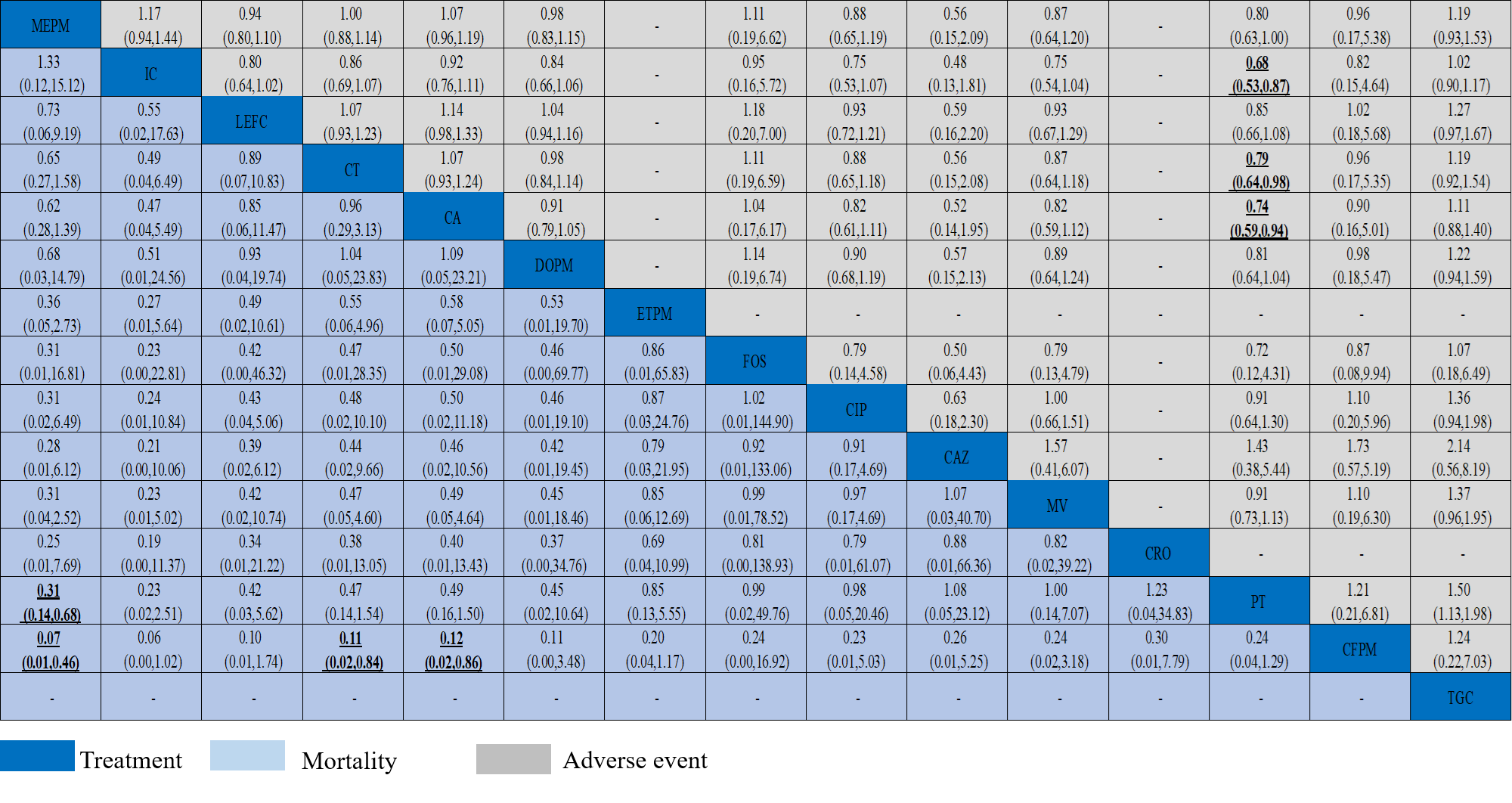


**Appendix 8**

**Figure S13.** Forest plots showing relative risk with 95% confidence interval of clinical success with a sample size more than 30 in a random-effects model. cUTI: complex urinary tract infection; cIAI: complicated intra-abdominal infection; CA: ceftazidime/avibactam; CAR: carbapenems.


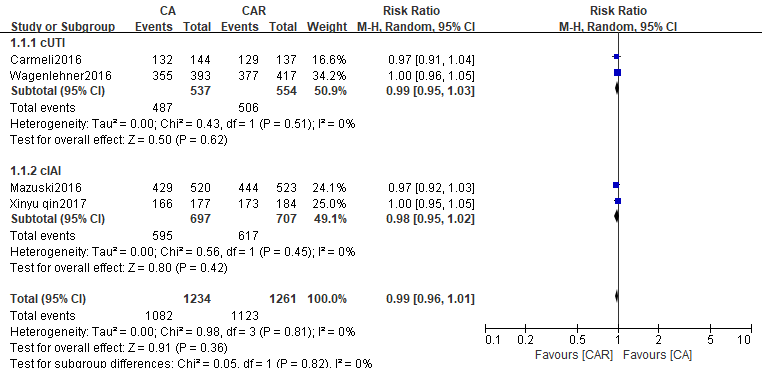


**Figure S14.** Forest plots showing relative risk with 95% confidence interval of microbiological success with a sample size more than 30 in a random-effects model. cUTI: complex urinary tract infection; cIAI: complicated intra-abdominal infection; CA: ceftazidime/avibactam; CAR: carbapenems.


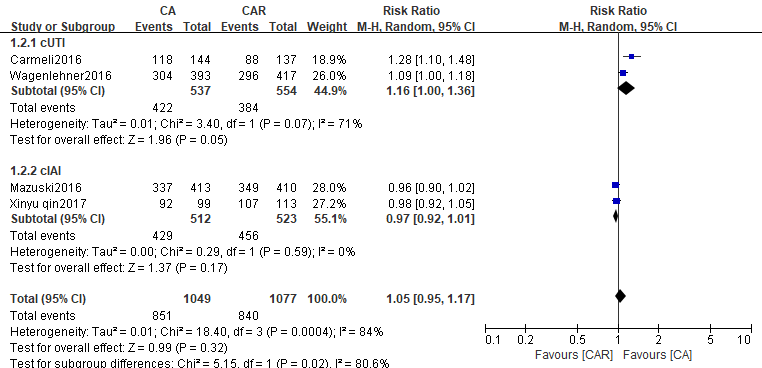


**Table S4** Surface under the cumulative ranking curve (SUCRA) ranking list for sample size ≥ 30

| Treatment | Clinical success | |  | Microbiological success | | Overall Rank^1^ |
| --- | --- | --- | --- | --- | --- | --- |
|  | SUCRA | Rank |  | SUCRA | Rank |  |
| Meropenem/vaborbactam | 60.6 | 5 |  | 83.9 | 2 | 1 |
| Meropenem | 83.1 | 1 |  | 53.4 | 6 | 2 |
| Imipenem/cilastatin | 60.7 | 4 |  | 65.5 | 5 | 3 |
| Ceftazidime/avibactam | 71.2 | 2 |  | 35.5 | 9 | 4 |
| Fosfomycin | 29.4 | 11 |  | 86.8 | 1 | 5 |
| Ceftolozane/tazobactam | 56.5 | 7 |  | 53 | 7 | 6 |
| Ceftriaxone | 60.3 | 6 |  | 43.9 | 8 | 7 |
| Tigecycline | 17 | 13 |  | 80.6 | 3 | 8 |
| Ciprofloxacin | 20.8 | 12 |  | 67.4 | 4 | 9 |
| Doripenem | 70.2 | 3 |  | 4.3 | 13 | 10 |
| Ertapenem | 48.9 | 8 |  | 34.3 | 10 | 11 |
| Levofloxacin | 38.2 | 9 |  | 11.4 | 12 | 12 |
| Piperacillin/tazobactam | 33.2 | 10 |  | 32.2 | 11 | 13 |
| Ceftazidime | - | - |  | - | - | - |

The orders were from best to worst.

^1^adding the order of clinical success and microbiological success together (Interventions with the same ranking would be re-ranked based on the sum of their SUCRA probabilities).

**Figure S15** Clinical success and microbiological success of antibiotics in studies with a sample size more than 30, drugs were sorted in the order of decreasing success. The clinical success results were compared from left to right, and the microbiological success results should be read from right to left (the results were expressed by relative risk with 95% confidence interval). Significant results were shown in bold and underlined. MEPM: meropenem, CA: ceftazidime/avibactam, DOPM: doripenem, IC: imipenem/cilastatin, MV: meropenem/vaborbactam, CRO: ceftriaxone, CT: ceftolozane/tazobactam, ETPM: ertapenem, LEFC: levofloxacin, PT: piperacillin/tazobactam, CIP: ciprofloxacin, TGC: tigecycline.


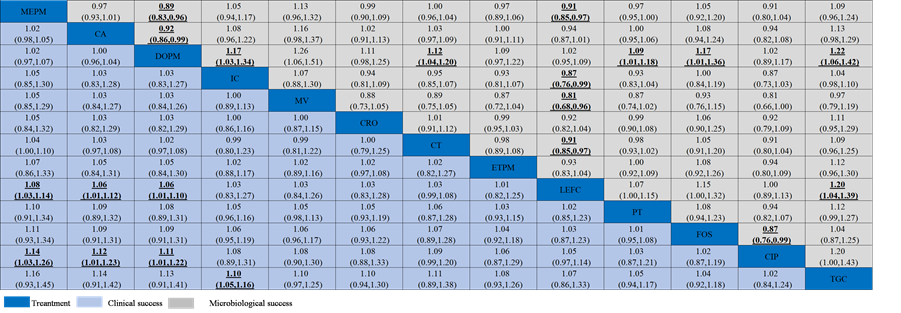


**Appendix 9**

**Table S5** Surface under the cumulative ranking curve (SUCRA) ranking list for sample size ≥100

| Treatment | Clinical success | |  | Microbiological success | | Overall Rank^1^ |
| --- | --- | --- | --- | --- | --- | --- |
|  | SUCRA | Rank |  | SUCRA | Rank |  |
| Meropenem/vaborbactam | 61.9 | 5 |  | 88.2 | 1 | 1 |
| Meropenem | 85.7 | 1 |  | 59.4 | 5 | 2 |
| Imipenem/cilastatin | 62.4 | 4 |  | 71.5 | 4 | 3 |
| Ceftazidime/avibactam | 72.3 | 2 |  | 37.7 | 8 | 4 |
| Ceftolozane/tazobactam | 56.2 | 6 |  | 56.5 | 6 | 5 |
| Fosfomycin | 31.0 | 9 |  | 73.2 | 3 | 6 |
| Tigecycline | 18.0 | 11 |  | 86.7 | 2 | 7 |
| Doripenem | 71.2 | 3 |  | 7.3 | 11 | 8 |
| Piperacillin/tazobactam | 35.8 | 8 |  | 38.5 | 7 | 9 |
| Levofloxacin | 36.5 | 7 |  | 13.0 | 10 | 10 |
| Ciprofloxacin | 19.0 | 10 |  | 18.0 | 9 | 11 |

The clinical success and microbiological success order were from best to worst.

^1^adding the order of clinical success and microbiological success together (Interventions with the same ranking would be re-ranked based on the sum of their SUCRA probabilities).

**Figure S16** Clinical success and microbiological success of antibiotics in studies with a sample size more than 100, drugs were sorted in the order of decreasing success. The clinical success results were compared from left to right, and the microbiological success results should be read from right to left (the results were expressed by relative risk with 95% confidence interval). Significant results were shown in bold and underlined. CA: ceftazidime/avibactam, MV: meropenem/vaborbactam, MEPM: meropenem, DOPM: doripenem, IC: imipenem/cilastatin, CAZ: ceftazidime, CFPM: cefepime, CIP: ciprofloxacin, PT: piperacillin/tazobactam, ETPM: ertapenem, LEFC: levofloxacin, FOS: fosfomycin, CT: ceftolozane/tazobactam, TGC: tigecycline, CRO: ceftriaxone.


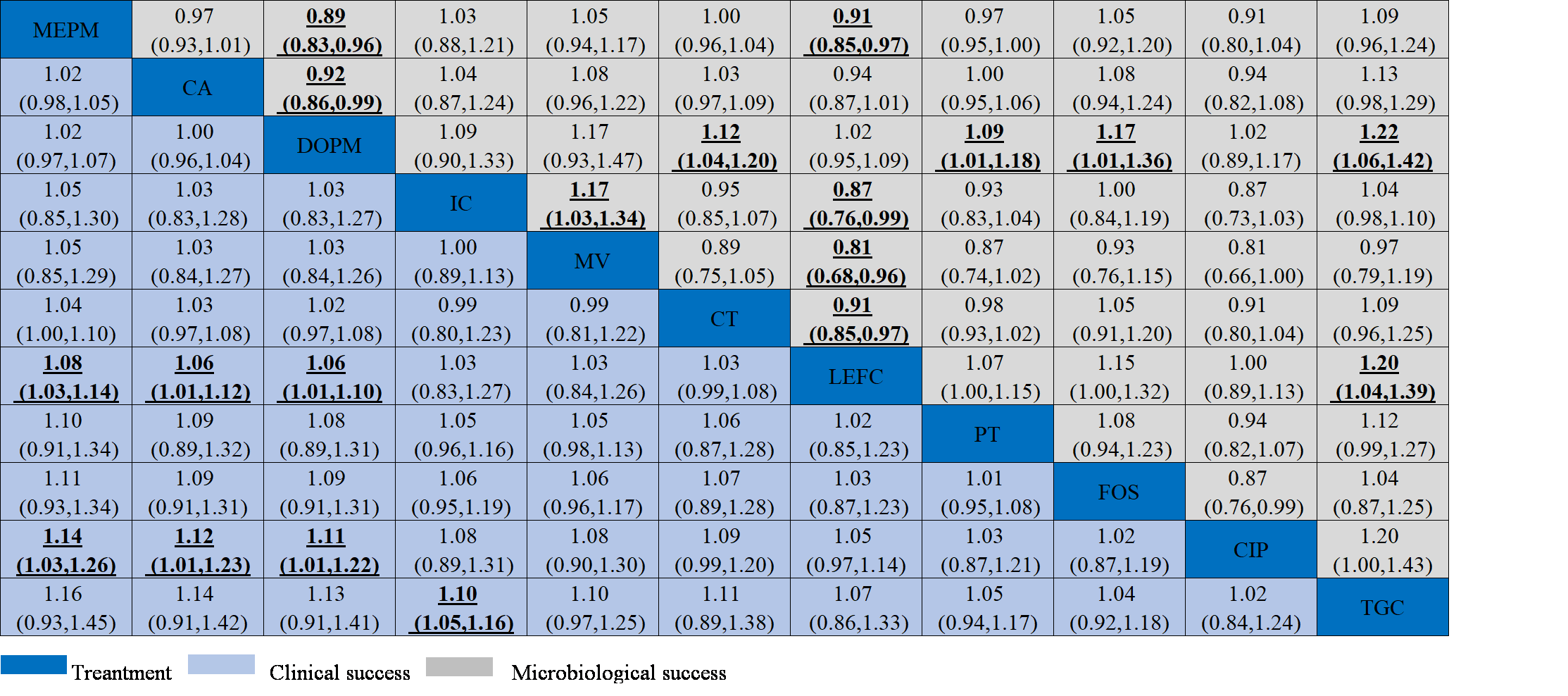


**References**

1. Huang W, Qiao F, Zhang Y, Huang J, Deng Y, Li J, et al. In-hospital Medical Costs of Infections Caused by Carbapenem-resistant Klebsiella pneumoniae. Clinical infectious diseases : an official publication of the Infectious Diseases Society of America. 2018;67(suppl_2):S225-s30.10.1093/cid/ciy642

2. Song Y, Tai JH, Bartsch SM, Zimmerman RK, Muder RR, Lee BY. The potential economic value of a Staphylococcus aureus vaccine among hemodialysis patients. Vaccine. 2012;30(24):3675-82.10.1016/j.vaccine.2012.03.031

3. Delate T, Coons SJ. The use of 2 health-related quality-of-life measures in a sample of persons infected with human immunodeficiency virus. Clinical infectious diseases : an official publication of the Infectious Diseases Society of America. 2001;32(3):E47-52.10.1086/318492

4. S.E. Geerlings CvN, M. van Buren, B.J. Knottnerus, E. E. Stobberingh, C.J. de Groot, J.M. Prins SWAB Guidelines for Antimicrobial Therapy of Complicated Urinary Tract Infections in Adults 2013.

5. Solomkin JS, Mazuski JE, Bradley JS, Rodvold KA, Goldstein EJ, Baron EJ, et al. Diagnosis and management of complicated intra-abdominal infection in adults and children: guidelines by the Surgical Infection Society and the Infectious Diseases Society of America. Surg Infect (Larchmt). 2010;11(1):79-109.10.1089/sur.2009.9930

6. Wagenlehner FM, Sobel JD, Newell P, Armstrong J, Huang X, Stone GG, et al. Ceftazidime-avibactam Versus Doripenem for the Treatment of Complicated Urinary Tract Infections, Including Acute Pyelonephritis: RECAPTURE, a Phase 3 Randomized Trial Program. Clinical infectious diseases : an official publication of the Infectious Diseases Society of America. 2016;63(6):754-62.10.1093/cid/ciw378

7. Kongnakorn T, Wagenlehner F, Falcone M, Tichy E, Di Virgilio R, Baillon-Plot N, et al. Cost-effectiveness analysis of ceftazidime/avibactam compared to imipenem as empirical treatment for complicated urinary tract infections. International journal of antimicrobial agents. 2019;54(5):633-41.10.1016/j.ijantimicag.2019.06.008

8. Nguyen CP, Dan Do TN, Bruggemann R, Ten Oever J, Kolwijck E, Adang EMM, et al. Clinical cure rate and cost-effectiveness of carbapenem-sparing beta-lactams vs. meropenem for Gram-negative infections: A systematic review, meta-analysis, and cost-effectiveness analysis. International journal of antimicrobial agents. 2019;54(6):790-7.10.1016/j.ijantimicag.2019.07.003

9. Wagenlehner FM, Umeh O, Steenbergen J, Yuan G, Darouiche RO. Ceftolozane-tazobactam compared with levofloxacin in the treatment of complicated urinary-tract infections, including pyelonephritis: a randomised, double-blind, phase 3 trial (ASPECT-cUTI). Lancet (London, England). 2015;385(9981):1949-56.10.1016/s0140-6736(14)62220-0

10. Vazquez JA, González Patzán LD, Stricklin D, Duttaroy DD, Kreidly Z, Lipka J, et al. Efficacy and safety of ceftazidime-avibactam versus imipenem-cilastatin in the treatment of complicated urinary tract infections, including acute pyelonephritis, in hospitalized adults: results of a prospective, investigator-blinded, randomized study. Current medical research and opinion. 2012;28(12):1921-31.10.1185/03007995.2012.748653

11. Kaye KS, Rice LB, Dane AL, Stus V, Sagan O, Fedosiuk E, et al. Fosfomycin for Injection (ZTI-01) Versus Piperacillin-tazobactam for the Treatment of Complicated Urinary Tract Infection Including Acute Pyelonephritis: ZEUS, A Phase 2/3 Randomized Trial. Clinical infectious diseases : an official publication of the Infectious Diseases Society of America. 2019;69(12):2045-56.10.1093/cid/ciz181

12. Harris PNA, Tambyah PA, Lye DC, Mo Y, Lee TH, Yilmaz M, et al. Effect of Piperacillin-Tazobactam vs Meropenem on 30-Day Mortality for Patients With E coli or Klebsiella pneumoniae Bloodstream Infection and Ceftriaxone Resistance: A Randomized Clinical Trial. Jama. 2018;320(10):984-94.10.1001/jama.2018.12163

13. Seo YB, Lee J, Kim YK, Lee SS, Lee JA, Kim HY, et al. Randomized controlled trial of piperacillin-tazobactam, cefepime and ertapenem for the treatment of urinary tract infection caused by extended-spectrum beta-lactamase-producing Escherichia coli. BMC infectious diseases. 2017;17(1):404.10.1186/s12879-017-2502-x

14. Qin X, Tran BG, Kim MJ, Wang L, Nguyen DA, Chen Q, et al. A randomised, double-blind, phase 3 study comparing the efficacy and safety of ceftazidime/avibactam plus metronidazole versus meropenem for complicated intra-abdominal infections in hospitalised adults in Asia. International journal of antimicrobial agents. 2017;49(5):579-88.10.1016/j.ijantimicag.2017.01.010

15. Mazuski JE, Gasink LB, Armstrong J, Broadhurst H, Stone GG, Rank D, et al. Efficacy and Safety of Ceftazidime-Avibactam Plus Metronidazole Versus Meropenem in the Treatment of Complicated Intra-abdominal Infection: Results From a Randomized, Controlled, Double-Blind, Phase 3 Program. Clinical infectious diseases : an official publication of the Infectious Diseases Society of America. 2016;62(11):1380-9.10.1093/cid/ciw133

16. Solomkin J, Hershberger E, Miller B, Popejoy M, Friedland I, Steenbergen J, et al. Ceftolozane/Tazobactam Plus Metronidazole for Complicated Intra-abdominal Infections in an Era of Multidrug Resistance: Results From a Randomized, Double-Blind, Phase 3 Trial (ASPECT-cIAI). Clinical infectious diseases : an official publication of the Infectious Diseases Society of America. 2015;60(10):1462-71.10.1093/cid/civ097

17. Park DW, Peck KR, Chung MH, Lee JS, Park YS, Kim HY, et al. Comparison of ertapenem and ceftriaxone therapy for acute pyelonephritis and other complicated urinary tract infections in Korean adults: a randomized, double-blind, multicenter trial. Journal of Korean medical science. 2012;27(5):476-83.10.3346/jkms.2012.27.5.476

18. Ceran N, Mert D, Kocdogan FY, Erdem I, Adalati R, Ozyurek S, et al. A randomized comparative study of single-dose fosfomycin and 5-day ciprofloxacin in female patients with uncomplicated lower urinary tract infections. Journal of infection and chemotherapy : official journal of the Japan Society of Chemotherapy. 2010;16(6):424-30.10.1007/s10156-010-0079-z

19. Klausner HA, Brown P, Peterson J, Kaul S, Khashab M, Fisher AC, et al. A trial of levofloxacin 750 mg once daily for 5 days versus ciprofloxacin 400 mg and/or 500 mg twice daily for 10 days in the treatment of acute pyelonephritis. Current medical research and opinion. 2007;23(11):2637-45.10.1185/030079907x233340

20. Wells WG, Woods GL, Jiang Q, Gesser RM. Treatment of complicated urinary tract infection in adults: combined analysis of two randomized, double-blind, multicentre trials comparing ertapenem and ceftriaxone followed by appropriate oral therapy. The Journal of antimicrobial chemotherapy. 2004;53 Suppl 2:ii67-74.10.1093/jac/dkh208

21. Ponce-de-León A, López-Meneses M, Sifuentes-Osornio J. Cefepime versus ceftazidime for the treatment of serious bacterial infections. Diagnostic microbiology and infectious disease. 1999;35(4):263-8.10.1016/s0732-8893(99)00085-1

22. Chang SC, Fang CT, Hsueh PR, Liu CJ, Sheng WH, Hsieh SM, et al. Efficacy and safety of cefepime treatment in Chinese patients with severe bacterial infections: in comparison with ceftazidime treatment. International journal of antimicrobial agents. 1998;10(3):245-8.10.1016/s0924-8579(98)00040-5

23. Sifuentes-Osornio J, Macías A, Amieva RI, Ramos A, Ruiz-Palacios GM. Intravenous ciprofloxacin and ceftazidime in serious infections. A prospective, controlled clinical trial with third-party blinding. The American journal of medicine. 1989;87(5a):202s-5s.10.1016/0002-9343(89)90059-4

24. Richard GA, Klimberg IN, Fowler CL, Callery-D'Amico S, Kim SS. Levofloxacin versus ciprofloxacin versus lomefloxacin in acute pyelonephritis. Urology. 1998;52(1):51-5.10.1016/s0090-4295(98)00160-5

25. Jimenez-Cruz F, Jasovich A, Cajigas J, Jiang Q, Imbeault D, Woods GL, et al. A prospective, multicenter, randomized, double-blind study comparing ertapenem and ceftriaxone followed by appropriate oral therapy for complicated urinary tract infections in adults. Urology. 2002;60(1):16-22.10.1016/s0090-4295(02)01664-3

26. Erasmo AA, Crisostomo AC, Yan LN, Hong YS, Lee KU, Lo CM. Randomized comparison of piperacillin/tazobactam versus imipenem/cilastatin in the treatment of patients with intra-abdominal infection. Asian journal of surgery. 2004;27(3):227-35.10.1016/s1015-9584(09)60039-7

27. Preheim LC, Childs SJ, Rajfer J, Bittner MJ. Randomized, double-blind comparison of cefepime and ceftazidime therapy for urinary tract infection. Current Therapeutic Research - Clinical and Experimental. 1995(No.8):729-37

28. Tomera KM, Burdmann EA, Reyna OG, Jiang Q, Wimmer WM, Woods GL, et al. Ertapenem versus ceftriaxone followed by appropriate oral therapy for treatment of complicated urinary tract infections in adults: results of a prospective, randomized, double-blind multicenter study. Antimicrobial agents and chemotherapy. 2002;46(9):2895-900.10.1128/aac.46.9.2895-2900.2002

29. Kaye KS, Bhowmick T, Metallidis S, Bleasdale SC, Sagan OS, Stus V, et al. Effect of Meropenem-Vaborbactam vs Piperacillin-Tazobactam on Clinical Cure or Improvement and Microbial Eradication in Complicated Urinary Tract Infection: The TANGO I Randomized Clinical Trial. Jama. 2018;319(8):788-99.10.1001/jama.2018.0438

30. Fomin P, Koalov S, Cooper A, Babinchak T, Dartois N, De Vane N, et al. The efficacy and safety of tigecycline for the treatment of complicated intra-abdominal infections - the European experience. Journal of chemotherapy (Florence, Italy). 2008;20 Suppl 1:12-9.10.1179/joc.2008.20.Supplement-1.12

31. Naber KG, Llorens L, Kaniga K, Kotey P, Hedrich D, Redman R. Intravenous doripenem at 500 milligrams versus levofloxacin at 250 milligrams, with an option to switch to oral therapy, for treatment of complicated lower urinary tract infection and pyelonephritis. Antimicrobial agents and chemotherapy. 2009;53(9):3782-92.10.1128/aac.00837-08

32. Carmeli Y, Armstrong J, Laud PJ, Newell P, Stone G, Wardman A, et al. Ceftazidime-avibactam or best available therapy in patients with ceftazidime-resistant Enterobacteriaceae and Pseudomonas aeruginosa complicated urinary tract infections or complicated intra-abdominal infections (REPRISE): a randomised, pathogen-directed, phase 3 study. The Lancet Infectious diseases. 2016;16(6):661-73.10.1016/s1473-3099(16)30004-4
